# Developmental perfluorooctane sulfonate (PFOS) exposure alters gene expression in nucleus accumbens and prefrontal cortex and impairs cognition in rats: A transcriptomic and mediation analysis

**DOI:** 10.1016/j.ecoenv.2025.119648

**Published:** 2026-01-06

**Authors:** Shiwen Li, Hongxu Wang, Ana C. Maretti-Mira, Tomas K. D. Manea, Shaun Y. Kim, Lida Chatzi, Jesse A. Goodrich, Tanya L. Alderete, Nathan Young, Ruth I. Wood, Max T. Aung

**Affiliations:** a Department of Population and Public Health Sciences, University of Southern California Keck School of Medicine, Los Angeles, California 90032, United States; b Department of Public Health Sciences, Thompson School of Social Work & Public Health, University of Hawaii at Manoa, Honolulu, HI 96822, United States; c Department of Medicine, University of Southern California Keck School of Medicine, Los Angeles, California 90033, United States; d Department of Integrative Anatomical Sciences, University of Southern California Keck School of Medicine, Los Angeles, California 90033, United States; e Department of Environmental Health and Engineering, Johns Hopkins Bloomberg School of Public Health, Baltimore, MD 21218, United States

**Keywords:** PFOS, Transcriptomics, Brain, Neurodevelopment

## Abstract

Growing evidence suggests that developmental exposure to perfluorooctanesulfonic acid (PFOS) is linked to neurobehavioral outcomes. Pregnant female rats were exposed to PFOS (15 mg/L) or Tween vehicle through drinking water until the offspring were weaned at three weeks of age. As adults, cognitive flexibility and impulsive decision-making were assessed in 8 PFOS-exposed and 8 vehicle-exposed rats using extradimensional set-shifting and delay discounting tasks, respectively. Cognitive flexibility was measured by the number of trials required to reach the criterion, while impulsive decision-making was quantified as the area under the curve (AUC) of the percent preference for the large reward lever (% CHL), response omissions (% omit), and response latency (in second) at delays of 0, 15, 30, and 45 s. Brain tissues from the nucleus accumbens, prefrontal cortex, and hippocampus were extracted for bulk RNA sequencing. Differential gene expression analysis and gene set enrichment analysis were performed. Mediation analysis was performed to assess the mediated effect of DEGs in the associations between PFOS and neurobehavioral tests. We identified 62 differentially expressed genes (DEGs) in the nucleus accumbens, 34 in the hippocampus, and 59 in prefrontal cortex tissues due to PFOS exposure. We also found DEGs, including *NAT8F2*, *AC080157.1*, *ABCG3*, and *ENSRNOG00000063145* mediated between PFOS and neurobehavioral assessments. Pathways that were associated with both PFOS exposure and neurobehavioral outcomes (% CHL and % omit) included extracellular matrix-receptor interaction, focal adhesion, and glutathione metabolism in the nucleus accumbens. Developmental PFOS exposure may alter gene expression in the nucleus accumbens and prefrontal cortex and was associated with impaired cognitive flexibility and impulsive decision-making. These exploratory findings highlight potential pathways, including ECM-receptor interaction and glutathione metabolism, that warrant further validation.

## 1. Introduction

Per- and polyfluoroalkyl substances (PFAS) are synthetic chemicals widely used in industrial processes and consumer products due to their resistance to water, oil, and heat (Gaines, 2023). Among these, perfluorooctane sulfonate (PFOS) is a legacy PFAS that remains pervasive in both the environment and the human body (Abunada et al., 2020). PFOS is particularly concerning because it resists metabolic breakdown, resulting in prolonged bioaccumulation (Rosato et al., 2024). Although overall PFAS levels have declined over recent years, PFOS continues to be the most frequently detected PFAS in human blood, with average concentrations ranging from 4.3 to 30.4 µg/L in the National Health and Nutrition Examination Survey (NHANES) (Sonnenberg et al., 2023).

Humans are primarily exposed to PFOS through contaminated drinking water, food, and consumer products (Wee and Aris, 2023). Epidemiological studies have linked PFOS exposure to several adverse health outcomes, including low birth weight, altered inflammatory responses, liver dysfunction, and an increased risk of certain cancers (Wee and Aris, 2023). Importantly, growing evidence also supports the neurotoxic potential of PFOS as this chemical can cross the blood-brain barrier and accumulate in brain tissues in animals and humans (Hong et al., 2024; Wang et al., 2018; Xie et al., 2024a; Yu et al., 2020). Elevated PFOS concentrations in regions such as the brain stem, hippocampus, hypothalamus, pons/medulla, and thalamus are of particular concern because these areas are critical for maintaining normal behavioral and cognitive functions (Baizer, 2021; Brown-Leung and Cannon, 2022; Quian Quiroga, 2023; Van Drunen and Eckel-Mahan, 2021). Although the precise mechanisms underlying PFOS-induced neurotoxicity remain poorly understood, several pathways have been proposed, including glial activation, mitochondrial dysfunction, alterations in neurotransmission, and indirect effects via hepatic encephalopathy and the gut-brain axis (Bharal et al., 2024).

Beyond these findings, epidemiological investigations have reported associations between PFOS exposure and neurodevelopmental disorders such as attention-deficit/hyperactivity disorder (ADHD), autism spectrum disorders (ASD), and cognitive deficits, with PFOS consistently implicated across studies (Currie et al., 2024; Yao et al., 2023). Recent research further suggests a link between PFOS exposure and Alzheimer’s disease (Delcourt et al., 2024; Lu et al., 2024). Complementing these human studies, animal research has shown that both acute and developmental exposure to PFOS can lead to significant neurobehavioral and neurochemical alterations. For instance, zebrafish exposed to PFOS display impaired swim bladder inflation, abnormal tail ventroflexion, and hyperactivity even at nonteratogenic concentrations (Gaballah et al., 2020). In rat models, prenatal and perinatal exposure to PFAS mixtures results in neonatal growth attenuation, delayed reflex development, and persistent hyperactivity (Marchese et al., 2024), while studies in frogs have noted reductions in brain dopamine levels following PFOS exposure (Foguth et al., 2019). Additionally, PFOS-induced changes in the transcriptome in zebrafish have been suggested as a contributing factor to its developmental toxicity (Rericha et al., 2024); however, investigations into tissue-specific transcriptomic alterations, particularly within the brain, remain limited.

Understanding the transcriptomic changes in distinct brain regions is crucial because each region plays a unique role in behavior and neurological function, and PFOS appears to accumulate differentially across these regions (Brown-Leung and Cannon, 2022). Moreover, transcriptomic profiling may help identify early molecular events and regulatory networks that precede overt neurobehavioral deficits. Such insights could illuminate the underlying mechanisms of PFOS-induced neurotoxicity and aid in developing biomarkers for early detection and risk assessment, ultimately informing regulatory policies and therapeutic strategies.

In the present study, we address this gap by analyzing differential gene expression in three key brain regions including the nucleus accumbens, hippocampus, and prefrontal cortex from the offspring of rats exposed to PFOS during pregnancy and neurobehavioral outcomes in the offspring rats. These regions were specifically chosen due to their distinct functional roles: the nucleus accumbens is central to motivation and action, the hippocampus is critical for learning and memory (including spatial memory), and the prefrontal cortex is essential for executive function and working memory (Genon et al., 2018). By dissecting the brain region-specific transcriptomic responses to PFOS exposure, our study aims to provide a more nuanced understanding of its neurotoxic effects and to identify potential molecular targets for intervention.

## 2. Method

### 2.1. Animals

Timed-pregnant Long-Evans rats (Envigo, Indianapolis, IN) were received at embryonic day 12 (E12) and delivered pups naturally. Animals were kept on a 14:10 light/dark cycle at 25 ± 2°C with ad libitum access to food and water until behavioral training. Male and female pups from at least three litters (n = 8/group) were used to control for litter effects, and in the present study, we only selected males for brain RNA sequencing to account for potential sex-specific effects. After weaning at three weeks, pups were pair-housed with same-sex partners under a reversed light cycle and trained/tested five days per week under dim light. Growth was managed at 1–2 g/day for training. Procedures were approved by the University of Southern California (USC) Institutional Animal Care and Use Committee, following the Guide for the Care and Use of Laboratory Animals (2011) (Guide for the Care and Use of Laboratory Animals, 2011).

### 2.2. PFOS Treatment

Pregnant females were exposed to PFOS (15 mg/L, CAS 2795–39–3, ≥ 98 % purity, Sigma-Aldrich) with 0.004 % Tween 20 in drinking water (n = 6) or the Tween vehicle (n = 6) from embryonic day 12 (E12) until weaning at postnatal day 21 (P21). Although pups are not exposed to PFOS after weaning, this combined pre- and post-natal PFOS exposure impairs spatial memory in adolescent rats measured in the Morris water maze (Wang et al., 2015). In addition, a systematic review documented several rat studies of PFOS exposure with similar dose ranges (0.12–20 mg/L) (Toxicological Profile for Perfluoroalkyls, 2021). This dose equates to a human equivalent of 2.4 mg/L for a 60 kg person.

A single human-relevant dose (15 mg/L) was selected to enable exploratory transcriptomic screening. This concentration falls within the range reported in prior rat studies of neurobehavioral alterations. Although additional dose groups were not included, these findings provide critical tissue-specific gene expression signatures to inform future studies that use varying doses to assess dose–response relationships and lower environmentally relevant concentrations.

### 2.3. Set shifting

Male rats were tested for extradimensional set-shifting from a direction cue task (DCT) to a visual cue task (VCT) using a modified protocol from Wallin and Wood (2015) (Wallin and Wood, 2015). Rats were first trained to press levers to receive sucrose pellets, habituated over daily 90-trial sessions until they met the criterion of fewer than five omissions. During testing, rats completed daily sessions of 64 trials (20 s/trial) involving illuminated stimulus lights and lever presses, with performance criteria set at eight consecutive correct responses. In DCT, rats had to ignore stimulus lights and press a consistent lever, whereas in VCT, the correct response was under the illuminated light. Rats had 10 days to reach the set-shifting criterion, or they were assigned a score of 640 trials.

### 2.4. Delay discounting

Rats underwent delay discounting testing as per Fahrenkopf et al. (2021) (Fahrenkopf et al., 2021). They were trained to discriminate between levers delivering one pellet versus three pellets, progressing to delayed rewards once they preferred the large reward lever (>80 % in 48 trials). Daily sessions of 48 trials included blocks with increasing delays (0, 15, 30, 45 s) on the large reward lever. Performance over 15 days was assessed, focusing on percent preference for the large reward (% CHL), response omissions (% omit), and response latency (in second). We then calculated area under the curve for each variable over the increasing delays. Thus, for %CHL, a higher AUC reflects better delay tolerance (less impulsivity), whereas, for % omissions and response latency, a higher AUC reflects poorer neurobehavioral performance (greater omissions or slower responding).

### 2.5. RNA Sequencing

Brain region-specific tissues were extracted at 129 days (18 weeks). Frozen tissues were transported on dry ice and stored at −80°C freezer until analysis. In male rats, RNA was extracted from the nucleus accumbens, hippocampus, and prefrontal cortex, followed by quality assessment using an RNA Pico Bioanalyzer. Quantification of bulk RNA transcripts was performed with Illumina Stranded Total RNA Prep, and Ligation with Ribo-Zero Plus. Library alignment, quality control, and gene annotation were conducted using Partek Flow. Briefly, we trimmed low-quality reads from both 5’ and 3’ ends based on quality scores. We used the STAR aligner to map these reads to a reference sequence. We then assigned each read to a known gene using a Quantification to Annotation model developed by Partek Flow. We then filtered genes that were identified in less than or equal to 10 samples for each region and gene counts were subsequently normalized using the median ratio method (Zyprych-Walczak et al., 2015).

### 2.6. Differential Gene Expression Analysis and Pathway Analysis

We conducted differential gene expression analysis comparing PFOS-exposed rats to controls in each tissue. Differentially expressed genes (DEGs) were identified using an exploratory threshold of crude p < 0.01 and |fold-change| > 1.5, followed by calculation of Benjamini–Hochberg false discovery rate (FDR)-adjusted p-values. Both adjusted and unadjusted values are reported to balance discovery sensitivity and false-discovery control.

Fold change in figures was expressed in the log_2_ scale for better visualization. Gene set enrichment analysis was performed using the Kyoto Encyclopedia of Genes and Genomes (KEGG) database. Significant pathways were identified at a FDR of p < 0.05. We used a strict threshold for pathway analysis only since we used a relatively relaxed p-value cut-off for the differential gene expression analysis to select the genes included in the pathway analysis.

To link molecular changes to neurobehavior tests, we next repeated the same differential-expression and GSEA workflow for each neurobehavioral measure (set shifting and delay discounting) separately in each tissue treating performance on each test as the “exposure” variable in place of PFOS. Finally, in our meet-in-the-middle analysis, we intersected the sets of significant KEGG pathways from the PFOS comparison and from each behavioral association within the same tissue. Pathways common to both PFOS exposure and behavioral performance within the same tissue were identified as candidate mechanistic links between transcriptomic alterations and neurobehavioral outcomes.

### 2.7. Effect of PFOS on Outcomes

We evaluated the effect of PFOS on each neurobehavioral outcome using Welch two-sample *t*-test, reporting the mean difference with 95 % confidence intervals (CI) and using a p-value of 0.05 as the threshold for statistical significance.

### 2.8. Mediation Analysis

Lastly, we identified overlapping DEGs between PFOS exposure and each of the neurobehavioral tests within each brain tissue. For the identified DEGs, we then conducted mediation analysis to assess the mediating role of differentially expressed genes. We first conducted mediation analysis assuming each of the DEGs mediated the effect of PFOS on neurobehavioral tests independently (via “*mediation*” R package). Then we assumed that each of DEGs mediated the effect of PFOS in parallel (via “*CMAverse*” R package). We obtained total indirect/mediated effect of DEGs from each of the mediation analysis.

**Fig. 1.**
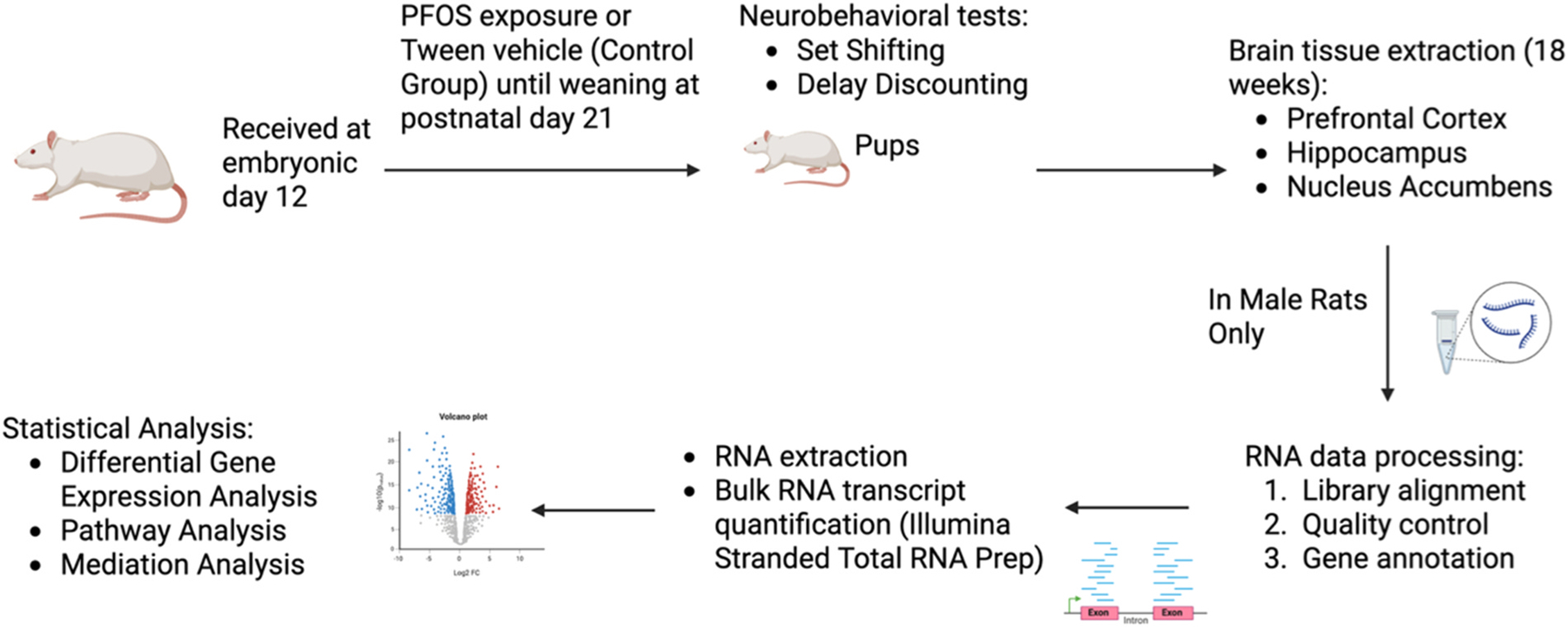
Overview of the study design. * Created in BioRender. Li, S. (2025) https://BioRender.com/119ayof.

An overview of the study design is presented in Fig. 1.

## 3. Results

**Fig. 2.**
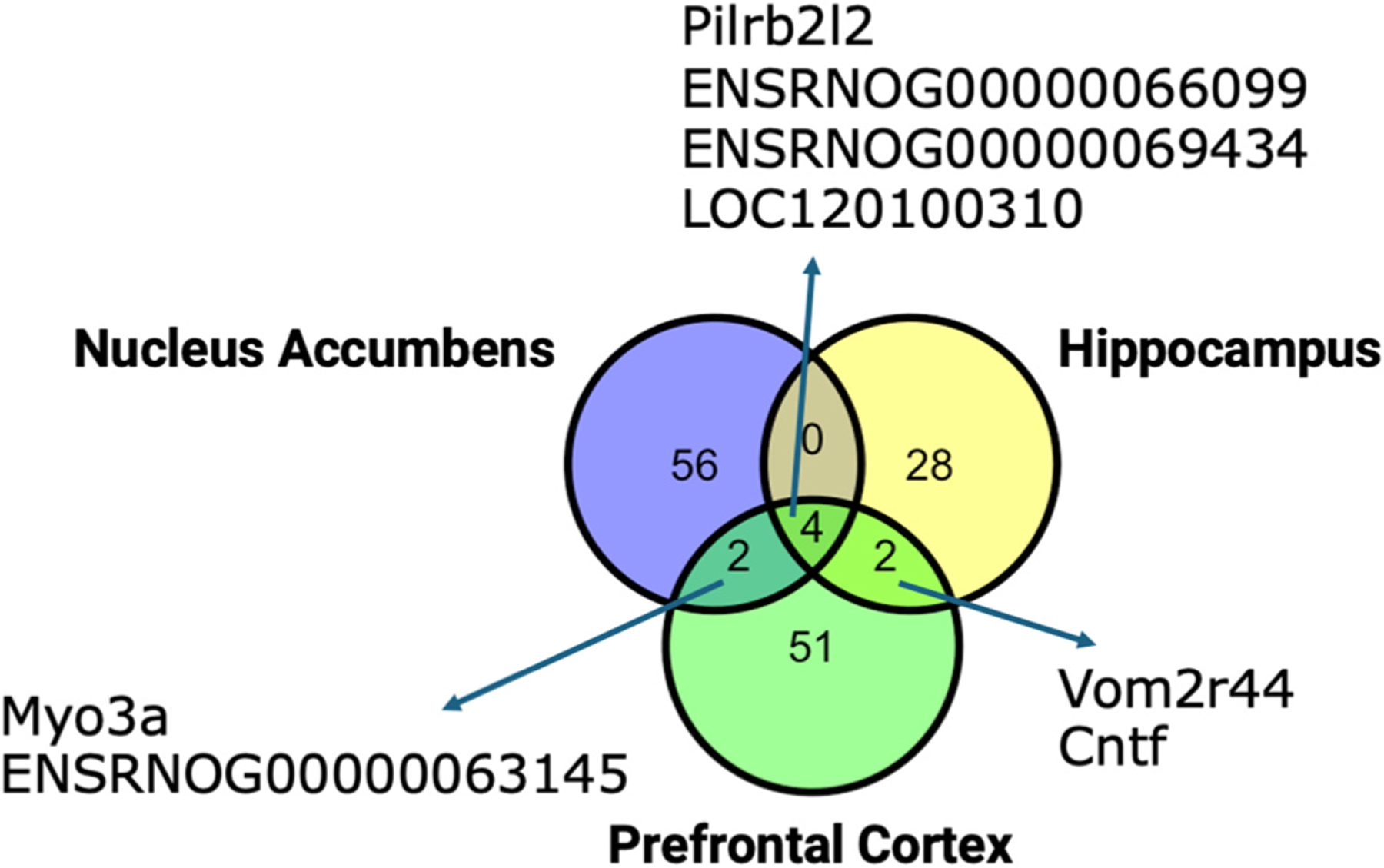
Venn diagrams depicting the distribution of differentially expressed genes (DEGs) in nucleus accumbens, hippocampus, and prefrontal cortex tissues due to PFOS exposure. *DEGs were identified using unadjusted p < 0.01 and | fold-change| > 1.5. **After applying multiple-testing correction using an adjusted p-value threshold of 0.05, one DEG (ITGB4) in the nucleus accumbens, two DEGs (ENSRNOG00000070065 and ENSRNOG00000069434) in the hippocampus, and one DEG (Pilrb2l2) in the prefrontal cortex remained statistically significant.

Transcriptome profiling was performed on tissues from three brain regions: 12 nucleus accumbens samples (6 PFOS, 6 control), 11 hippocampus samples (4 PFOS, 7 control), and 14 prefrontal cortex samples (7 PFOS, 7 control). After preprocessing, 16,242 genes were aligned to transcripts included in the analysis. Differential expression analysis identified 62 genes in the nucleus accumbens, 34 genes in the hippocampus, and 59 genes in the prefrontal cortex as significantly differentially expressed. Among these, four genes were common to all tissues; two genes were shared between the nucleus accumbens and prefrontal cortex, and two were common between the hippocampus and prefrontal cortex (see Fig. 2). After applying multiple-testing correction using an adjusted p-value threshold of 0.05, one DEG (*ITGB4*) in the nucleus accumbens, two DEGs (*ENSRNOG00000070065* and *ENSRNOG00000069434*) in the hippocampus, and one DEG (*Pilrb2l2*) in the prefrontal cortex remained statistically significant (see Supplemental Table 1).

**Fig. 3.**
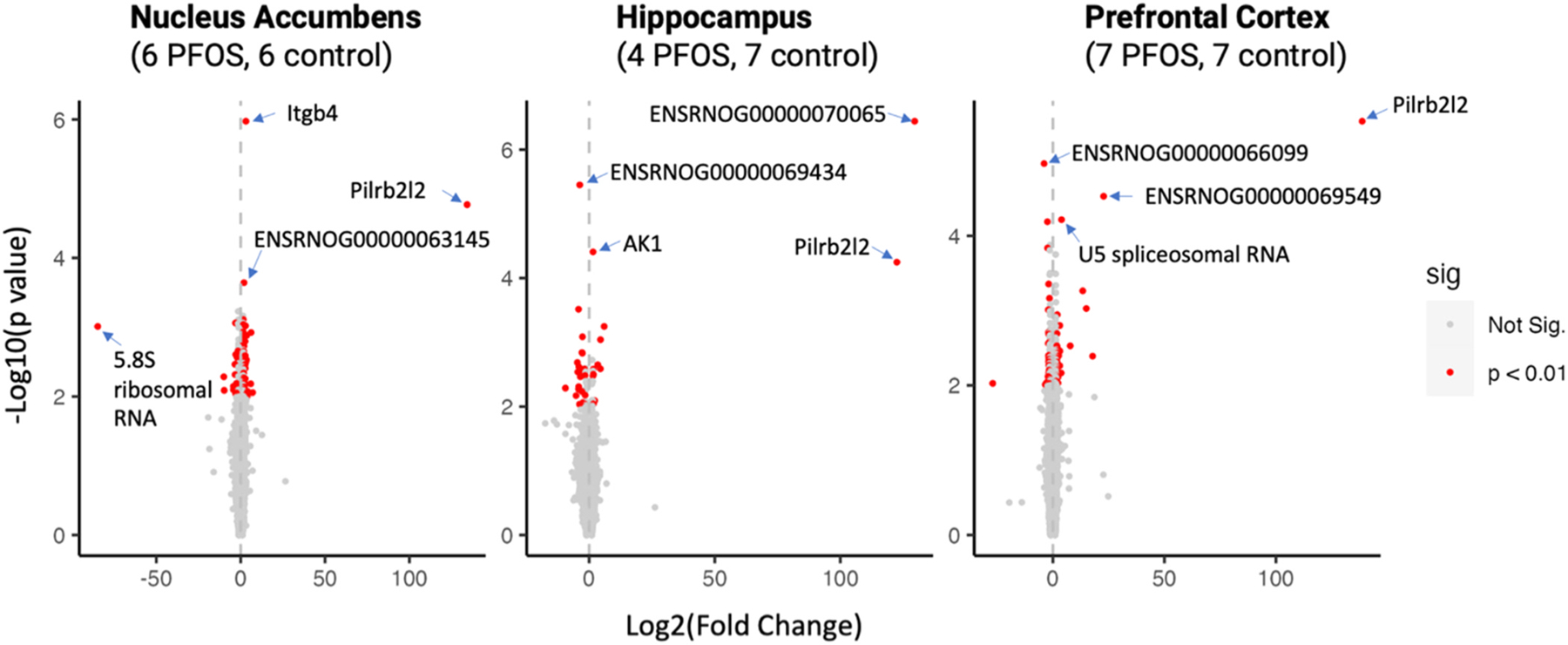
Volcano plot of differentially expressed genes due to PFOS exposure by brain tissue type. *Significant differentially expressed genes (DEGs) were identified based on crude p < 0.01 and a fold change > 1.5 or < −1.5. Fold change was expressed in the log2 scale for better visualization. **Crude p value was -log10 transformed for better visualization. ***After applying multiple-testing correction using an adjusted p-value threshold of 0.05, one DEG (ITGB4) in the nucleus accumbens, two DEGs (ENSRNOG00000070065 and ENSRNOG00000069434) in the hippocampus, and one DEG (Pilrb2l2) in the prefrontal cortex remained statistically significant.

Within the nucleus accumbens tissues, shown in Fig. 3, PFOS exposure dysregulated 55 protein-coding genes, 3 long non-coding RNAs (lncRNAs), 2 ribosomal RNAs (rRNAs), 1 mitochondrial rRNA, and 1 small nucleolar RNA (snoRNA). Of the protein-coding genes, 13 were downregulated and 42 were upregulated. Notably, *ITGB4* (fold change: 3.10 [1.97, 4.88], adjusted p-value=0.02) and *Pilrb2l2* (fold change: 134.20 [14.40, 1250.73], adjusted p-value=0.14) were among the most affected. Pathway analysis (FDR<0.05) in nucleus accumbens tissues revealed 8 significantly enriched pathways, including ECM-receptor interaction, protein digestion and absorption, focal adhesion, PI3K-Akt signaling, amoebiasis, AGE-RAGE signaling in diabetic complications, human papillomavirus infection, and relaxin signaling, with genes involved these pathways including *COL1A1, COL1A2, COL4A5, COL4A6, COL6A3, ITGB4, MYC*, and *SLC9A3*.

In the hippocampus tissues, PFOS exposure dysregulated 29 protein-coding genes (20 protein-coding genes downregulated and nine upregulated), four lncRNAs, and one snoRNA. The top candidates included *ENSRNOG00000069434*, a predicted membrane protein; *AABR07051450.1*, a gene encoding for a putative ATP-binding protein; and *Pilrb2l2*, with respective fold changes and adjusted p-values of −3.76 [−2.15, −6.57] (0.03), 1.55 [1.26, 1.91] (0.21), and 122.50 [11.79, 1272.21] (0.23). No pathways reached significance at an FDR< 0.05 in the hippocampus tissues.

**Fig. 4.**
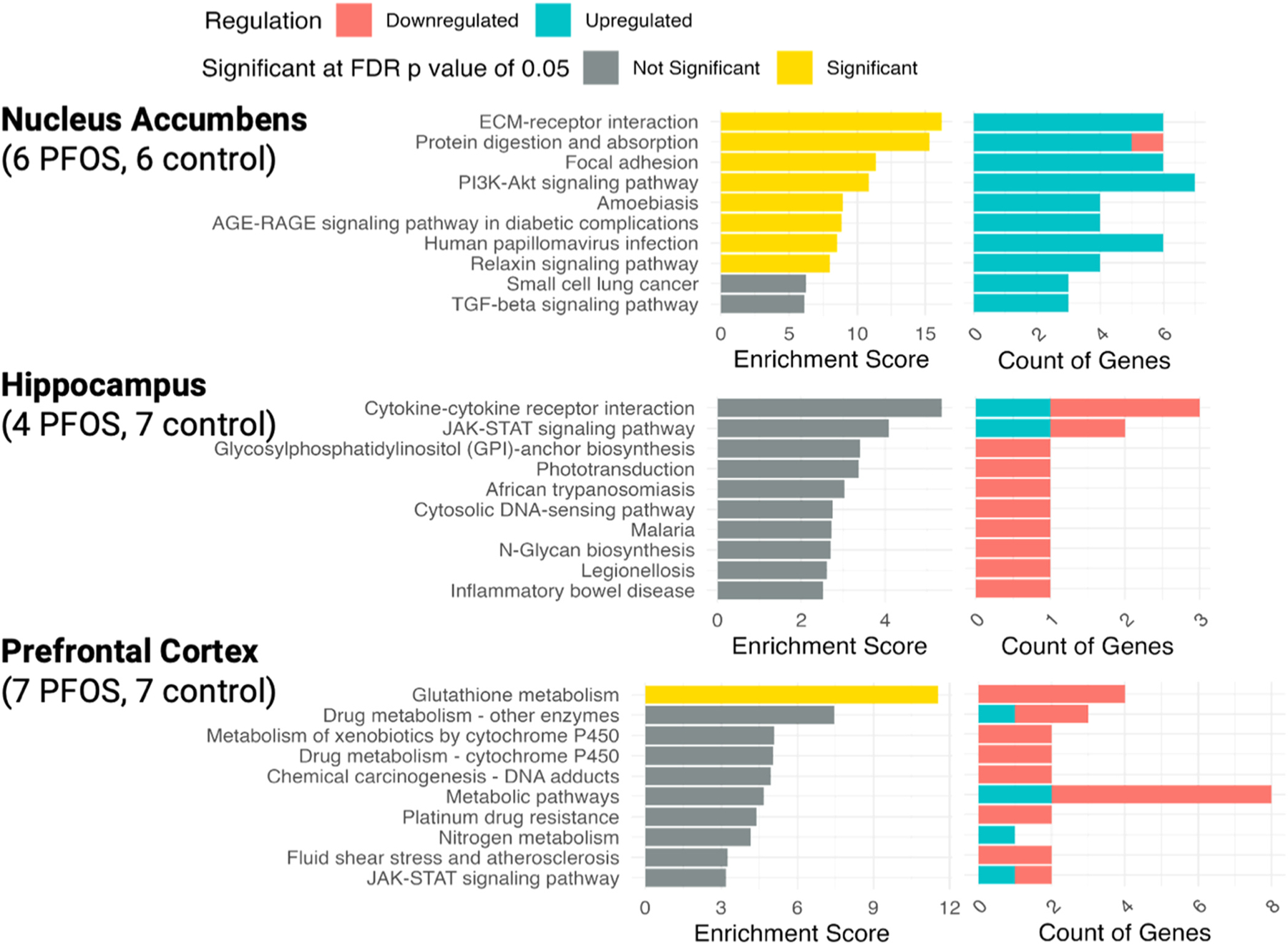
Pathway analysis by brain tissues comparing PFOS-exposed rats versus controls using Kyoto Encyclopedia of Genes and Genomes (KEGG) based on differential gene expression analysis. *On the right panel, the upregulation of genes (colored by blue) and downregulation of genes (colored by red) for each of the pathways were extracted from the differential gene expression analysis. Significant differentially expressed genes (DEGs) were identified based on crude p < 0.01 and a fold change > 1.5 or < −1.5. **Significant pathways were identified at a false discovery rate (FDR) of p < 0.05 and were colored in yellow on the left panel. An enrichment score quantifies how much a set of genes (representing a pathway or function) is over-represented at the top or bottom of a ranked list of genes, indicating enrichment or depletion of that pathway.

Within the prefrontal cortex tissues, PFOS exposure resulted in the dysregulation of 41 protein-coding genes (24 were downregulated and 17 were upregulated), 4 lncRNAs, 5 spliceosomal RNAs (snRNAs), 3 pseudogenes, 3 snoRNAs, 1 Small Cajal body-specific RNA (scaRNA), and 1 rRNA. The most affected genes in the prefrontal cortex included *Pilrb2l2*, *ENSRNOG00000069549*, and *Vom2r44*, with fold changes of 138.63 [17.55, 1095.16] (FDR p = 0.05), 1.55 [5.25, 98.52] (FDR p = 0.16; not statistically significant), and −2.45 [−3.81, −1.58] (FDR p = 0.21; not statistically significant), respectively. Pathway enrichment analysis (FDR p < 0.05) in the prefrontal cortex revealed that glutathione metabolism was the sole significantly enriched pathway, involving genes such as *GPT6*, *GSTA1*, *GSTM4*, and *NAT8F1*. Full summary statistics for the differential gene expression and pathway analyses associated with PFOS are available in Supplemental Tables 1 and 2. Pathway analysis results for PFOS exposure are presented in Fig. 4.

**Fig. 5.**
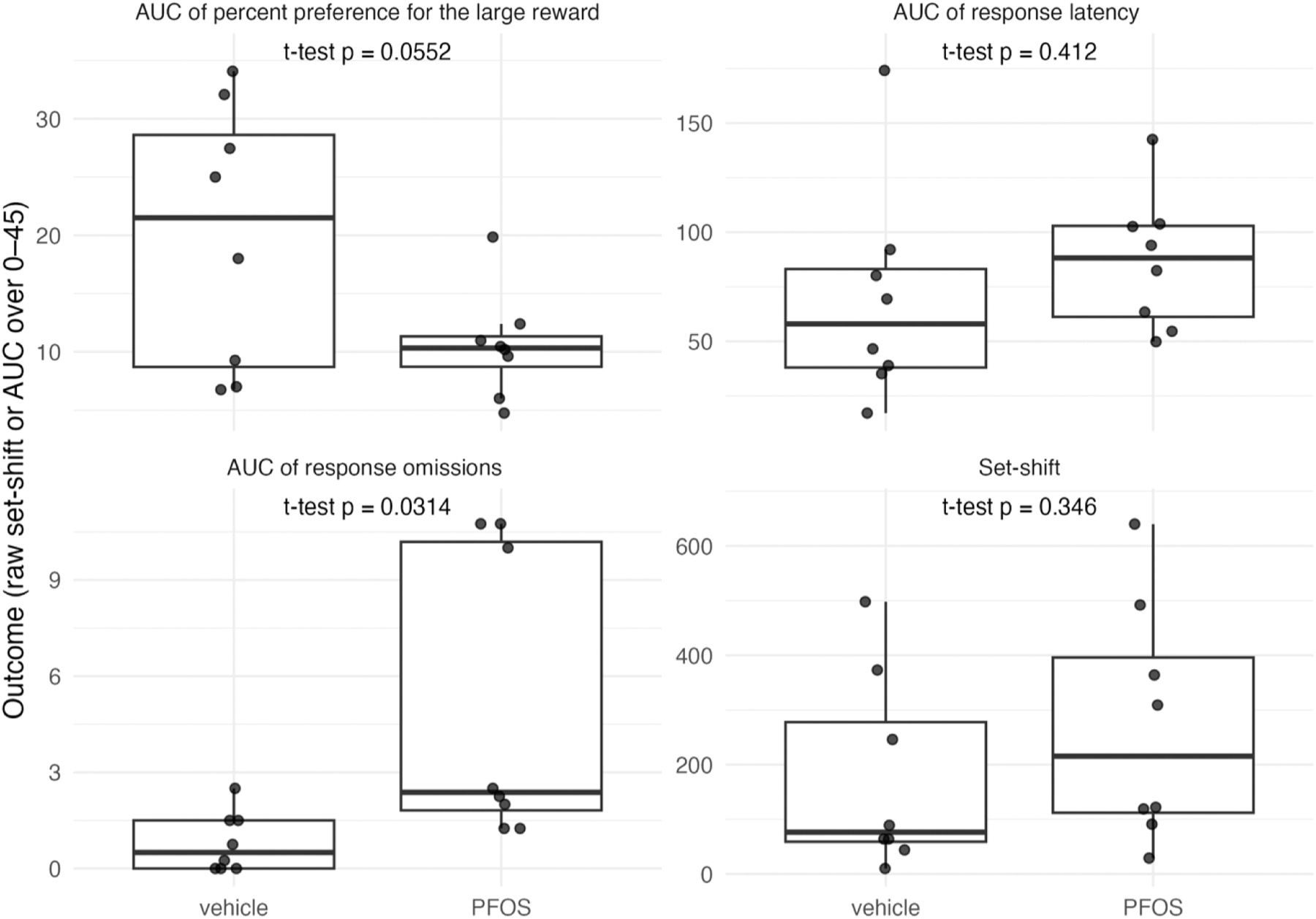
Comparison of neurobehavioral outcome between PFOS exposed and vehicle exposed rat.

Based on two-sample Welch *t*-tests comparing PFOS-exposed and vehicle groups, PFOS exposure was not associated with set-shifting performance (mean difference [PFOS — vehicle] = 97.3 trials, 95 % CI −117.3–311.8, p = 0.35). In the delay-discounting task (0–45 s), PFOS (vs vehicle) was associated with a borderline significant lower percentage of choices for the large reward (mean difference = −9.4 %age points, 95 % CI −19.1–0.26, p = 0.055) and was associated with more omissions (mean difference = 4.3 points, 95 % CI 0.50–8.07, p = 0.031). The difference in response latency was not statistically significant (mean difference = 17.4 s, 95 % CI −27.4–62.3, p = 0.41). Full summary statistics are included in Table S3, and the distribution of outcomes by PFOS exposure group is shown in Fig. 5.

As shown in Table S4, across the three brain regions, we observed a total of 14 pathways associated with set shifting (8 in nucleus accumbens, 5 in prefrontal cortex and 1 in hippocampus), 15 with response omission (3 in nucleus accumbens, 11 in prefrontal cortex, 1 in hippocampus), 26 with choosing large reward (13 in nucleus accumbens, 7 in prefrontal cortex, and 6 in hippocampus), 40 with response latency (16 in nucleus accumbens, 12 in prefrontal cortex, 12 in hippocampus).

**Fig. 6.**
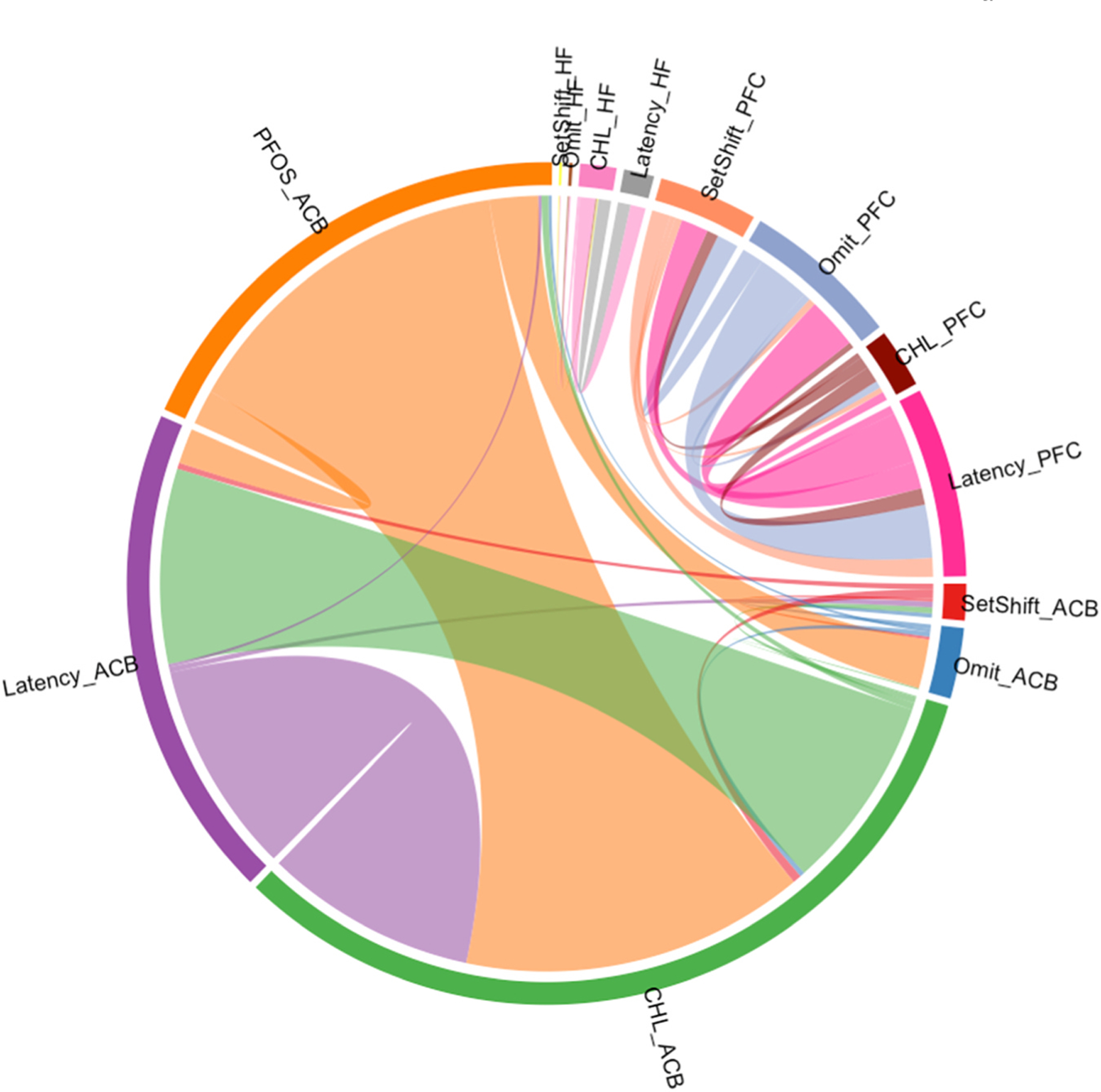
Within-Tissue Overlap of Enriched Pathways for PFOS Exposure and Neurobehavioral Measures in Nucleus Accumbens, Hippocampus, and Prefrontal Cortex. *ACB: nucleus accumbens; PFC: prefrontal cortex; HF: hippocampus; CHL: percent preference for the large reward; Omit: response omissions; SetShift: set shifting; Lines are weighted by adjusted p value and pathway enrichment score. **The overlapping pathways are both overlapping pathways between the PFOS exposure enriched pathway and neurobehavioral measures, as well as between different neurobehavioral measures within the same tissue.

Among these pathways, only PFOS-associated pathways within nucleus accumbens overlapped with neurobehavioral test-associated pathways which included ECM-receptor interaction (under-enriched in choosing large reward and over-enriched in response omission), protein digestion and absorption (over-enriched in response latency), and focal adhesion (under-enriched in choosing large reward). See Fig. 6.

When assessing the overlapping DEGs, we found that 4 DEGs overlapped between PFOS and choosing large reward (2 in nucleus accumbens and 2 in prefrontal cortex), 9 overlapped between PFOS and response omission (2 in nucleus accumbens and 7 in prefrontal cortex) (See Table S5).

**Fig. 7.**
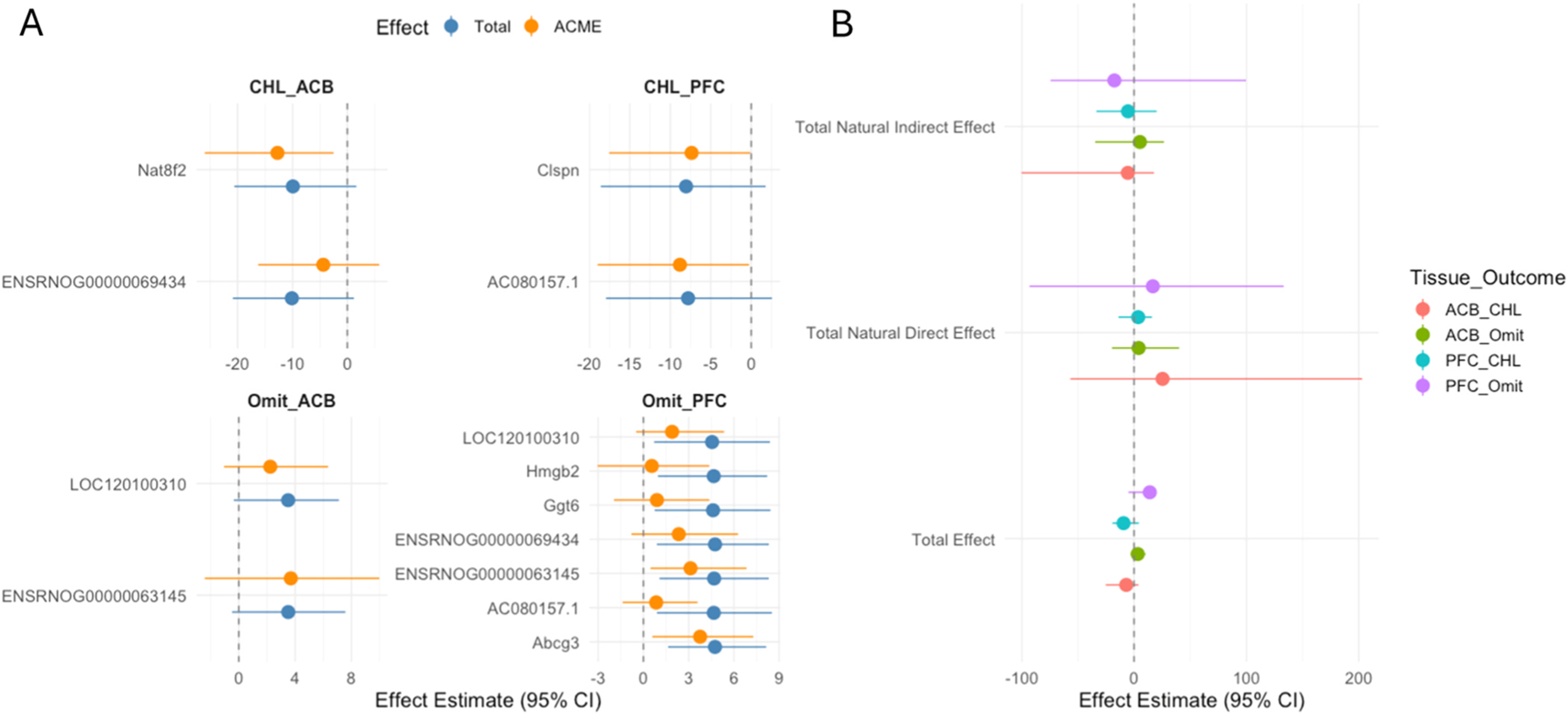
Within-tissue mediation analysis of the differentially expressed genes in the effect of PFOS on neurobehavioral outcomes assuming A) independent mediated effect and B) parallel mediated effect. *ACME: average causal mediated effect; Tota: total effect; ACB: nucleus accumbens; PFC: prefrontal cortex; CHL: percent preference for the large reward; Omit: response omissions.

When we conducted mediation analysis assuming mediated effect of these DEGs was independent, we found that Nat8f2 mediated about 100 % of the total effect of PFOS on preference of choosing large reward in nucleus accumbens (total and mediated effect beta [95 %CI]: −9.95 [−20.61, 1.66] and −12.76 [−26, −2.51]) while AC080157.1 mediated about 100 % in prefrontal cortex (−7.8 [−17.95, 2.55] and −8.81 [−18.98, −0.26]). Abcg3 mediated about 80 % of PFOS effect on response omission in prefrontal cortex (4.74 [1.64, 8.14] and 3.76 [0.59, 7.29]) while ENSRNOG00000063145 for 65 % (3.13 [0.47, 6.83] and 4.69 [1.07, 8.31]). However, when we assume the mediated effects of DEGs were parallel, we did not find any overall significant total natural indirect effect. Effect estimates and 95 % CI were plotted in Fig. 7 and full summary statistics are available in Tables S6 and S7.

## 4. Discussion

In the present exploratory study, we are the first study, to our knowledge, that showed that developmental exposure to PFOS was linked with transcriptomic alterations primarily in the nucleus accumbens and prefrontal cortex tissues, with fewer changes detected in the hippocampus. However, given the exploratory nature of the study, our results should be interpreted with caution. Multiple pathways were dysregulated in nucleus accumbens tissues, including those related to ECM-receptor interaction, protein digestion and absorption, focal adhesion, and the PI3K-Akt signaling pathway—pathways that have been implicated in neurodegenerative diseases (Rosh et al., 2024). In prefrontal cortex tissues, we observed a notable impact on glutathione metabolism, a critical pathway for the detoxification of reactive oxygen species (ROS) (R. Dringen et al., 2000). We also showed that several genes may mediate the effect of PFOS on neurobehavioral outcomes including *NAT8F2*, *AC080157.1*, *ABCG3*, and *ENSRNOG00000063145* in nucleus accumbens and prefrontal cortex as well as pathways that were linked to both PFOS exposure and % omit and CHL in the nucleus accumbens, including ECM-receptor interaction, protein digestion and absorption, and focal adhesion.

Overall, we identified modest DEGs in different brain regions due to PFOS exposure. There are several reasons that might explain the results. Because PFOS exposure occurred prenatally and during lactation rather than through direct administration, the resulting perturbations in offspring brain tissue were expected to be subtle. PFOS has a long biological half-life and primarily acts through chronic, low-level endocrine and metabolic dysregulation rather than acute cytotoxicity, which typically produces smaller changes in gene expression amplitude (Fenton et al., 2021; Rickard et al., 2022). Moreover, our region-specific profiling of the nucleus accumbens, prefrontal cortex, and hippocampus prioritized biological specificity over statistical power, as each region contains distinct neuronal and glial populations whose cell–type–specific responses may be diluted when analyzed at the bulk-tissue level (Kozlenkov et al., 2014). The developmental timing of exposure and tissue collection may also have limited the magnitude of detectable transcriptional effects, since perinatal neurogenesis, synaptic remodeling, and hormonal maturation can buffer transient disturbances, producing functional and behavioral changes without large-scale differential expression (Semple et al., 2013; Tau and Peterson, 2010). Finally, inter-individual variation in maternal transfer, PFOS metabolism, and regional brain uptake may contribut to variability and dampened gene expression signals meeting statistical thresholds. Together, these factors suggest that the subtle, region-specific transcriptional changes observed here may nevertheless represent biologically meaningful molecular adaptations underlying the neurobehavioral alterations identified in PFOS-exposed offspring. The findings of this study provide a critical foundation of brain tissue specific gene expression signals for exploring PFOS induced neurotoxicity in future studies with long-term PFOS exposure, increased sample size, and additional brain region tissue gene expression signals.

### 4.1. Extracellular Matrix and PI3K-Akt signaling pathway in nucleus accumbens tissues

In our study of nucleus accumbens tissue, we observed upregulation of several collagen family genes (*COL1A1, COL1A2, COL4A5, COL4A6, and COL6A3*), as well as *ITGB4* and *MYC*. These genes are integral to both the PI3K-Akt signaling pathway and extracellular matrix (ECM)- receptor interaction. Notably, the *ITGB4* gene was upregulated in nucleus accumbens tissue, and to our knowledge, no previous study on PFAS exposure has identified this gene. *ITGB4* encodes an integrin that is critical for cell-cell and cell-matrix adhesion, and previous studies have linked its dysregulation to various cancers, including prostate, colorectal, lung cancers, and glioma (P. Chen et al., 2024; Li et al., 2019; Wilkinson et al., 2020; Wu et al., 2019; Zhang et al., 2024). More, the overexpression of *ITGB4* has been suggested as a prognostic biomarker for lung adenocarcinoma brain metastasis and lower grade glioma (P. Chen et al., 2024; Zhang et al., 2024). Future mechanistic studies should aim to identify the exact role of *ITGB4* in PFOS-induced neurotoxicity.

Our results further suggest that PFOS may induce restructuring of the ECM in the nucleus accumbens tissues via the overexpression of collagen family genes. These collagen genes encode the proteins that form the basic components of the ECM, which provides structural stability in various tissues including the brain (Chu, 2011). Alterations in the ECM have been implicated in various brain-related diseases (Soles et al., 2023), suggesting that collagen plays an important role in both neurodevelopment and neurodegeneration (Wareham et al., 2024). Supporting this notion, previous studies in mice with mixed anxiety/depression-like states have shown that multiple collagen family genes can be either upregulated or downregulated in different brain regions (Smagin et al., 2019).

Furthermore, in the present study, ECM-receptor interaction was also linked with preference of choosing large reward and response omission which aligned with the previous studies (Bhuiyan et al., 2022; Reichardt and Tomaselli, 1991; Soles et al., 2023). Preference for the large reward measures the impulsivity and response omission measures roughly the sustained attention/motivational drive which are partly regulated by nucleus accumbens (a neural interface between motivation and action) (Salgado and Kaplitt, 2015). Our study adds to the evidence of a potential mechanistic link between PFOS exposure and changes in reward system and motivation through ECM.

The PI3K-Akt signaling pathway, which regulates cellular proliferation, growth, and survival, is activated by both cellular stimuli and toxicants (Martini et al., 2014). Dysregulation of this pathway has been implicated in numerous human diseases, including neurodevelopmental and neurodegenerative disorders (Rai et al., 2019; Wang et al., 2017). Growing evidence indicates that PFOS exerts its effects via the PI3K-Akt signaling pathway, as demonstrated by in vitro studies (in colorectal cancer and granulosa cell line) where PFOS has been shown to both activate and inhibit this pathway (Gao et al., 2024; F. Li et al., 2024). More specifically, PFOS appears to mediate its neurotoxic effects through the PI3K-Akt signaling pathway; for instance, one study demonstrated that this pathway plays a critical role in PFOS-induced inflammatory activation of astrocytes and subsequent IL-1β secretion (Chen et al., 2018a). Furthermore, PFOS has been shown to promote activation of the PI3K-Akt signaling pathway in human brain microvascular endothelial cells (HBMEC), which are major components of the blood-brain barrier (Wang et al., 2011). Taken together, these findings suggest that PFOS can traverse the blood-brain barrier and induce neuroinflammation via the PI3K-Akt signaling pathway.

It is important to note, however, that excessive activation of the Pl3k-Akt signaling pathway in nucleus accumbens tissues may contribute to neurobehavioral disorders, such as substance abuse disorders (Neasta et al., 2011). The nucleus accumbens region is critical for regulating reward and pleasure behaviors, and evidence indicates that activation of the Pl3k-Akt pathway in this region can enhance heroin-seeking behaviors following withdrawal in rats (Zhu et al., 2021), whereas its inhibition can attenuate alcohol use and abuse disorders (Neasta et al., 2011).

### 4.2. Glutathione metabolism in prefrontal cortex tissue

In the prefrontal cortex tissues, we identified the downregulation of several key genes involved in glutathione metabolism, including *GGT6*, *GSTA1*, *GSTM4*, and *NAT8F1*. In the brain, glutathione metabolism is essential for the detoxification of oxidative stressors, such as reactive oxygen species (ROS) (Ralf Dringen et al., 2000). Depletion of brain glutathione has been observed in human tissues as well as in in vitro studies of aging and neurodegenerative diseases such as Alzheimer’s and Parkinson’s disease (Dwivedi et al., 2020). Additionally, glutathione is hypothesized to be involved in neurodevelopmental diseases such as autism spectrum disorder. Oxidative conversion of glutathione to glutathione disulfide can lead to proteotoxic stress or glutamate excitotoxicity, ultimately resulting in neuronal death (Bjørklund et al., 2021).

The prefrontal cortex is responsible for complex cognitive behaviors including decision-making, reasoning, personality expression, working memory, and spatial memory (El-Baba and Schury, 2024). Altered glutathione metabolism has been observed in rat models of major depression (Yang et al., 2013) and decreased glutathione levels have been reported in the prefrontal cortex of patients with psychiatric disorders (Gawryluk et al., 2011) and psychosis (Xin et al., 2016). These observations align well with our findings of reduced glutathione metabolism. Moreover, human metabolomics studies using peripheral blood have frequently identified PFAS-associated dysregulation of glutathione metabolism, including in studies of PFOS exposure (Y. Chen et al., 2024; India-Aldana et al., 2023; Prince et al., 2023; Rhee et al., 2023). In addition, PFAS is known to generate reactive oxygen species (ROS), and previous studies have shown elevated ROS levels in neuronal cells following PFOS exposure (S. Li et al., 2024). Thus, the excessive production of ROS induced by PFOS may dysregulate normal glutathione pathways, explaining the observed downregulation of the glutathione metabolism pathway. Although acute ROS exposure typically upregulates glutathione metabolism, prolonged oxidative stress can deplete glutathione pools and downregulate synthetic enzymes through feedback inhibition or mitochondrial dysfunction. The observed reduction in glutathione-related gene expression may therefore indicate compensatory exhaustion rather than activation (Jha et al., 2000; Marí et al., 2020; Ribas et al., 2014).

Our study further showed that *NAT8F2* can mediate almost 100 % of the total effect of PFOS on preference of choosing large reward (or impulsivity) in the nucleus accumbens of the rats. *NAT8F1* and *NAT8F2* are both orthologous to human *NAT8* gene. Some previous studies proposed *SHATI/NAT8L* as a novel inhibitory factor against methamphetamine dependence and showed *SHATI/NAT8L* KO mice significantly increased dopamine levels in the nucleus accumbens, increased dopamine turnover rate, and greater dopamine release after exposure to methamphetamine suggesting a strong link between *SHATI/NAT8L* KO mice or downregulation of *SHATI/NAT8L* and dopamine-related behavioral problems (Furukawa-Hibi et al., 2012; Toriumi et al., 2018, 2015). These previous studies strongly align with our observation of dysregulation of *NAT8F2* in the nucleus accumbens after PFOS exposure, associated with greater impulsivity. No previous studies have specifically assessed the role of *NAT8* family genes in the neurobehavioral outcomes and further study is needed.

### 4.3. Pilrb2l2 upregulation across all brain tissues

*Pilrb2l2*, which is orthologous to the human *PILRA* gene (Gaudet et al., 2011), was found to be upregulated across all three brain tissues examined in this study. Numerous studies have suggested that PILRA, which is driven by the activation of glial cells, plays a role in neuroinflammation and has been linked to Alzheimer’s disease and possibly Parkinson’s disease, with some reports even proposing its utility as a prognostic biomarker (Agostini et al., 2021; Li et al., 2023; Lopatko Lindman et al., 2022; Miller et al., 2020; Patel et al., 2018; Shi et al., 2023). If PFOS can influence the expression of *PILRA*, it may contribute to long-term impacts on the brain, potentially increasing the risk of neurodegenerative diseases. However, given the limited studies investigating PFOS and neurodegeneration in both human and animal models, further research is needed to confirm these findings.

Moreover, we observed a very high level of fold change in gene expression for *Pilrb2l2*. The large fold change was not sensitive to different normalization methods. This is mostly because there was very low expression among the control rats resulting in a seemingly large fold change. It is also possible that our tissue extract resulted in more glial cells among the PFOS-treated rats than the controls. Lastly, we cannot rule out potential measurement errors due to low input RNA.

### 4.4. Strengths and limitations

One of the strengths of our study is that we investigated a critical window of exposure, prenatal PFOS exposure, and its effects on transcriptomic changes and neurobehavioral outcomes in the brains of offspring. By examining different brain tissues representing distinct functions, we provide a more comprehensive view of the mechanisms underlying PFOS neurotoxicity and identified potential mechanism through our mediation analysis. Furthermore, the use of a human-relevant PFOS dosage enhances the translational value of our findings. However, our study also has limitations. We focused exclusively on male pups, which precludes any assessment of potential sex-specific effects of PFOS exposure.

It is important to note that our DEG results should be interpreted cautiously as exploratory, given that we used a more relaxed cut-off for significant DEGs. The relatively limited sample sizes may have contributed to reduced statistical power to detect sparse gene expression signals, and therefore, our pathway analysis results should be interpreted with caution.

In addition, we evaluated only a single PFOS dose, which prevents us from establishing a dose-response relationship. However, these findings establish a foundation for building future dose-response experiments. Finally, the use of bulk RNA sequencing did not allow us to account for cell-type-specific effects, which may be important for understanding the precise mechanisms of PFOS-induced neurotoxicity. Future studies can build on our findings to focus on hypothesis-driven testing of genes in cell-type-specific experiments.

Although PFOS concentrations were not measured in offspring brain tissue, prior work has confirmed that PFOS crosses the blood–brain barrier and accumulates in rodent and human brain at similar exposure levels (Brown-Leung and Cannon, 2022; Wang et al., 2018; Xie et al., 2024b). In addition to direct neurotoxic effects, PFOS may also exert systemic influences on brain function through peripheral pathways, such as endocrine disruption, metabolic perturbation, or immune-mediated signaling, which can secondarily impact neural processes (Chen et al., 2018b; Coperchini et al., 2021; Ehrlich et al., 2023; Fenton et al., 2021; Freire et al., 2023; Gaillard et al., 2025; Moyano et al., 2024).

Experimental protein validation was beyond this study’s scope. However, several DEGs identified here (e.g., ITGB4, GSTM4, NAT8F2) have been independently linked to PFAS-related oxidative and extracellular matrix signaling in other models (Barutcu et al., 2024; Beccacece et al., 2023; Chanas et al., 2002; Liang et al., 2023; Mei et al., n.d.; Ojo et al., 2021; Shi and Zhou, 2010; Yang et al., 2025). Future research should employ RT-qPCR, Western blot, and immunohistochemistry to validate these findings at the protein level.

## 5. Conclusion

To our knowledge, this is the first study to investigate brain-tissue-specific transcriptomic profiling following developmental exposure to PFOS in rats and the potential mediating effect of gene expression in the brain on the PFOS-induced neurobehavioral outcomes. Our findings suggest mechanisms that may explain PFOS neurotoxicity through alterations in the extracellular matrix and PI3K-Akt signaling pathway in nucleus accumbens tissues, disrupted glutathione metabolism in prefrontal cortex tissue, and the consistent upregulation of *Pilrb2l2* across all examined tissues. Future studies are needed to evaluate the effects of other PFAS, as well as PFAS mixtures, and to further assess the critical exposure windows required to induce changes in the brain transcriptome. Moreover, additional research should aim to confirm the links between PFAS exposure and neurological conditions through the pathways identified in our study. Lastly, it is also important to identify sensitive periods of exposure to PFOS (during pregnancy vs postnatal or childhood exposure).

## Supplementary Material

Supplemental Materials

Appendix A. Supporting information

Supplementary data associated with this article can be found in the online version at doi:10.1016/j.ecoenv.2025.119648.

## Data Availability

Data will be made available on request.
