# Supplementary material for: Developmental perfluorooctane sulfonate (PFOS) exposure alters gene expression in nucleus accumbens and prefrontal cortex and impairs cognition in rats: A transcriptomic and mediation analysis
Source: Ecotoxicol Environ Saf. Author manuscript; Available in PMC 2026 Jan 29. (PMC12854093; doi:10.1016/j.ecoenv.2025.119648)
Supplement: Supplemental Materials [file NIHMS2136135-supplement-Supplemental_Materials.docx]

| **Supplemental Table 1.** Differential gene expression analysis due to PFOS exposure in different brain tissues. | | | | | | | | | | | | | | |
| --- | --- | --- | --- | --- | --- | --- | --- | --- | --- | --- | --- | --- | --- | --- |
| **Tissue^1^** | **Gene ID** | **Transcript Biotype** | **Least Squares mean (PFOS)^2^** | **Least Squares mean (Control)^2^** | **Fold Change** | **Lower 95% CI** | **Upper 95% CI** | **P Value** | **FDR-Adjsuted P Value^3^** | **Chromosome** | **Start Position** | **Stop Position** | **Length** | **Strand** |
| ACB | Itgb4 | protein coding | 106.5240382 | 34.3986857 | 3.10 | 1.97 | 4.88 | 0.00 | 0.02 | 10 | 101206665 | 101243013 | 36348 | + |
| ACB | Pilrb2l2 | protein coding | 25.93843674 | 0.193286227 | 134.20 | 14.40 | 1250.73 | 0.00 | 0.14 | 12 | 18210535 | 18582203 | 371668 | - |
| ACB | ENSRNOG00000063145 | lncRNA | 116.4666327 | 59.37968658 | 1.96 | 1.37 | 2.81 | 0.00 | 1.00 | 1 | 114719526 | 114753075 | 33549 | - |
| ACB | Col4a5 | protein coding | 114.6037244 | 74.81890679 | 1.53 | 1.19 | 1.96 | 0.00 | 1.00 | X | 105118820 | 105322693 | 203873 | + |
| ACB | ENSRNOG00000066099 | lncRNA | 5.631508915 | 17.40203421 | -3.09 | -6.00 | -1.59 | 0.00 | 1.00 | 1 | 53816738 | 53818754 | 2016 | - |
| ACB | Thsd4 | protein coding | 173.6383312 | 109.3814393 | 1.59 | 1.21 | 2.09 | 0.00 | 1.00 | 8 | 60386875 | 61024996 | 638121 | - |
| ACB | Bmp7 | protein coding | 40.76505536 | 14.38064226 | 2.83 | 1.53 | 5.26 | 0.00 | 1.00 | 3 | 161516462 | 161716789 | 200327 | - |
| ACB | LOC120102591 | rRNA | 0.56252932 | 47.63753668 | -84.68 | -1185.31 | -6.05 | 0.00 | 1.00 | 4 | 494813 | 494966 | 153 | - |
| ACB | Adam1a | protein coding | 182.1358171 | 117.3023197 | 1.55 | 1.19 | 2.03 | 0.00 | 1.00 | 12 | 35017315 | 35020312 | 2997 | + |
| ACB | Slc26a7 | protein coding | 5.328609427 | 0.864263482 | 6.17 | 2.05 | 18.53 | 0.00 | 1.00 | 5 | 27887042 | 28021659 | 134617 | - |
| ACB | Cdh1 | protein coding | 40.88078635 | 11.58667921 | 3.53 | 1.63 | 7.62 | 0.00 | 1.00 | 19 | 34492371 | 34561776 | 69405 | + |
| ACB | Bgn | protein coding | 277.9147694 | 173.1324907 | 1.61 | 1.20 | 2.15 | 0.00 | 1.00 | X | 151197273 | 151209462 | 12189 | + |
| ACB | Cpxm2 | protein coding | 71.29134163 | 27.44852126 | 2.60 | 1.44 | 4.70 | 0.00 | 1.00 | 1 | 186836797 | 186947221 | 110424 | - |
| ACB | Myc | protein coding | 47.2977361 | 31.24103207 | 1.51 | 1.17 | 1.96 | 0.00 | 1.00 | 7 | 93593705 | 93598631 | 4926 | + |
| ACB | Fmo1 | protein coding | 60.83503788 | 30.69188545 | 1.98 | 1.29 | 3.05 | 0.00 | 1.00 | 13 | 75182176 | 75214648 | 32472 | - |
| ACB | Bphl | protein coding | 24.6051242 | 39.21944114 | -1.59 | -2.15 | -1.18 | 0.00 | 1.00 | 17 | 30799629 | 30837289 | 37660 | - |
| ACB | Aebp1 | protein coding | 298.6856842 | 160.3361104 | 1.86 | 1.25 | 2.78 | 0.00 | 1.00 | 14 | 80738892 | 80748878 | 9986 | + |
| ACB | Ddit4l | protein coding | 2.638599977 | 7.767435458 | -2.94 | -5.93 | -1.46 | 0.00 | 1.00 | 2 | 226128276 | 226131376 | 3100 | + |
| ACB | Angptl2 | protein coding | 37.86189958 | 14.20059403 | 2.67 | 1.41 | 5.05 | 0.00 | 1.00 | 3 | 16517420 | 16548179 | 30759 | + |
| ACB | Islr | protein coding | 48.07634433 | 18.44141208 | 2.61 | 1.40 | 4.86 | 0.00 | 1.00 | 8 | 58570777 | 58574131 | 3354 | - |
| ACB | ENSRNOG00000069434 | protein coding | 10.8440988 | 23.15129246 | -2.13 | -3.51 | -1.30 | 0.00 | 1.00 | 1 | 196028296 | 196037444 | 9148 | - |
| ACB | LOC120100310 | snoRNA | 14.34401909 | 4.222412757 | 3.40 | 1.52 | 7.61 | 0.00 | 1.00 | 1 | 110972130 | 110972223 | 93 | - |
| ACB | Tbx15 | protein coding | 31.70628113 | 13.16288217 | 2.41 | 1.35 | 4.31 | 0.00 | 1.00 | 2 | 186576676 | 186687664 | 110988 | + |
| ACB | Pwwp4 | protein coding | 7.583587848 | 2.408489095 | 3.15 | 1.47 | 6.73 | 0.00 | 1.00 | X | 151008123 | 151014217 | 6094 | + |
| ACB | Slc13a3 | protein coding | 232.8453006 | 113.0053004 | 2.06 | 1.27 | 3.33 | 0.00 | 1.00 | 3 | 154141872 | 154204607 | 62735 | - |
| ACB | S100a11 | protein coding | 7.927634327 | 2.789827348 | 2.84 | 1.42 | 5.70 | 0.00 | 1.00 | 2 | 179191715 | 179197045 | 5330 | + |
| ACB | Vim | protein coding | 447.2648317 | 270.7688135 | 1.65 | 1.18 | 2.31 | 0.00 | 1.00 | 17 | 76668647 | 76677188 | 8541 | + |
| ACB | LOC102554288 | protein coding | 202.910715 | 450.5751087 | -2.22 | -3.78 | -1.30 | 0.00 | 1.00 | 3 | 145508997 | 145532382 | 23385 | - |
| ACB | Cfap53 | protein coding | 131.7186138 | 198.5864249 | -1.51 | -1.98 | -1.15 | 0.00 | 1.00 | 18 | 67869855 | 67952319 | 82464 | + |
| ACB | ENSRNOG00000069297 | lncRNA | 1.865590424 | 6.338944806 | -3.40 | -7.71 | -1.50 | 0.00 | 1.00 | 4 | 125686239 | 125714069 | 27830 | + |
| ACB | Col1a1 | protein coding | 167.8524219 | 63.95531214 | 2.62 | 1.37 | 5.01 | 0.00 | 1.00 | 10 | 79883622 | 79900625 | 17003 | + |
| ACB | Slc22a8 | protein coding | 183.7695127 | 101.4370302 | 1.81 | 1.21 | 2.70 | 0.00 | 1.00 | 1 | 205498084 | 205517451 | 19367 | + |
| ACB | Myo3a | protein coding | 11.42319494 | 4.709421885 | 2.43 | 1.33 | 4.43 | 0.00 | 1.00 | 17 | 84543552 | 84759043 | 215491 | + |
| ACB | Fam180a | protein coding | 20.42632111 | 7.606646161 | 2.69 | 1.37 | 5.25 | 0.00 | 1.00 | 4 | 63992867 | 64008048 | 15181 | - |
| ACB | Btk | protein coding | 17.36397144 | 9.709766947 | 1.79 | 1.20 | 2.67 | 0.00 | 1.00 | X | 97722802 | 97761854 | 39052 | - |
| ACB | Serinc2 | protein coding | 2.475097027 | 8.208912259 | -3.32 | -7.64 | -1.44 | 0.00 | 1.00 | 5 | 142592309 | 142618365 | 26056 | - |
| ACB | Car9 | protein coding | 0.239639995 | 2.390733133 | -9.98 | -50.10 | -1.99 | 0.01 | 1.00 | 5 | 57763206 | 57769839 | 6633 | + |
| ACB | Coch | protein coding | 232.5193755 | 135.6046497 | 1.71 | 1.17 | 2.51 | 0.01 | 1.00 | 6 | 69031167 | 69045110 | 13943 | + |
| ACB | Col1a2 | protein coding | 346.4793667 | 130.6082514 | 2.65 | 1.33 | 5.29 | 0.01 | 1.00 | 4 | 32563381 | 32598868 | 35487 | + |
| ACB | Anxa2 | protein coding | 81.95308316 | 45.81343496 | 1.79 | 1.19 | 2.70 | 0.01 | 1.00 | 8 | 70105253 | 70141659 | 36406 | + |
| ACB | Nqo2 | protein coding | 39.57626725 | 22.55645938 | 1.75 | 1.17 | 2.62 | 0.01 | 1.00 | 17 | 30909187 | 30938321 | 29134 | - |
| ACB | Col6a3 | protein coding | 101.6430377 | 50.09953751 | 2.03 | 1.22 | 3.37 | 0.01 | 1.00 | 9 | 91361583 | 91439472 | 77889 | - |
| ACB | Kiss1r | protein coding | 11.69497856 | 5.581982082 | 2.10 | 1.23 | 3.57 | 0.01 | 1.00 | 7 | 9785135 | 9788794 | 3659 | - |
| ACB | Slc9a3 | protein coding | 4.642519726 | 11.55328326 | -2.49 | -4.80 | -1.29 | 0.01 | 1.00 | 1 | 29124674 | 29167418 | 42744 | - |
| ACB | Acy3 | protein coding | 27.53075252 | 17.7533051 | 1.55 | 1.13 | 2.13 | 0.01 | 1.00 | 1 | 201279851 | 201283176 | 3325 | + |
| ACB | Angptl7 | protein coding | 9.199715099 | 1.539459217 | 5.98 | 1.65 | 21.70 | 0.01 | 1.00 | 5 | 158932794 | 158937325 | 4531 | - |
| ACB | Aldh1a3 | protein coding | 20.85394074 | 7.320661563 | 2.85 | 1.34 | 6.07 | 0.01 | 1.00 | 1 | 119982277 | 120017437 | 35160 | - |
| ACB | ENSRNOG00000065408 | protein coding | 0.774000819 | 3.295839862 | -4.26 | -12.24 | -1.48 | 0.01 | 1.00 | 14 | 96067533 | 96074123 | 6590 | - |
| ACB | Agbl1 | protein coding | 14.47011034 | 6.557205982 | 2.21 | 1.24 | 3.93 | 0.01 | 1.00 | 1 | 130043970 | 130951639 | 907669 | + |
| ACB | Rtn4rl2 | protein coding | 28.48400978 | 79.75152295 | -2.80 | -5.94 | -1.32 | 0.01 | 1.00 | 3 | 69955169 | 69971489 | 16320 | - |
| ACB | Zic4 | protein coding | 31.47319922 | 13.58935918 | 2.32 | 1.25 | 4.28 | 0.01 | 1.00 | 8 | 91920015 | 91935369 | 15354 | + |
| ACB | Col4a6 | protein coding | 26.94126218 | 13.72832013 | 1.96 | 1.19 | 3.22 | 0.01 | 1.00 | X | 104766957 | 105117501 | 350544 | - |
| ACB | RGD1309651 | protein coding | 16.45239055 | 26.7013291 | -1.62 | -2.32 | -1.14 | 0.01 | 1.00 | 19 | 48660288 | 48686350 | 26062 | - |
| ACB | Ccn1 | protein coding | 9.343490918 | 40.06817261 | -4.29 | -12.57 | -1.46 | 0.01 | 1.00 | 2 | 234562408 | 234565485 | 3077 | - |
| ACB | LOC120101957 | rRNA | 1483.454557 | 14452.05561 | -9.74 | -52.62 | -1.80 | 0.01 | 1.00 | 3 | 2234750 | 2234903 | 153 | + |
| ACB | Popdc3 | protein coding | 4.41342355 | 1.047728089 | 4.21 | 1.44 | 12.29 | 0.01 | 1.00 | 20 | 48772462 | 48800594 | 28132 | + |
| ACB | Alx4 | protein coding | 4.843922383 | 0.687934774 | 7.04 | 1.64 | 30.28 | 0.01 | 1.00 | 3 | 79611719 | 79648261 | 36542 | + |
| ACB | Neurod2 | protein coding | 41.63857219 | 106.9827193 | -2.57 | -5.21 | -1.27 | 0.01 | 1.00 | 10 | 83324442 | 83327987 | 3545 | - |
| ACB | AY172581.24 | Mt rRNA | 33040.22779 | 98512.79677 | -2.98 | -6.78 | -1.31 | 0.01 | 1.00 | M | 1094 | 2665 | 1571 | + |
| ACB | RGD1562811 | protein coding | 5.63449391 | 1.368772238 | 4.12 | 1.42 | 11.96 | 0.01 | 1.00 | 8 | 51433558 | 51463336 | 29778 | - |
| ACB | Nat8f2 | protein coding | 45.22276695 | 16.47414908 | 2.75 | 1.28 | 5.89 | 0.01 | 1.00 | 4 | 118319988 | 118324396 | 4408 | - |
| ACB | ENSRNOG00000063076 | protein coding | 7.39913881 | 1.890988894 | 3.91 | 1.39 | 11.04 | 0.01 | 1.00 | 7 | 88803047 | 88804404 | 1357 | - |
| HF | ENSRNOG00000070065 | lncRNA | 307.2252059 | 2.370598327 | 129.60 | 19.90 | 843.91 | 0.00 | 0.01 | 2 | 20869527 | 20870650 | 1123 | + |
| HF | ENSRNOG00000069434 | protein coding | 9.044906508 | 33.98514415 | -3.76 | -6.57 | -2.15 | 0.00 | 0.03 | 1 | 196028296 | 196037444 | 9148 | - |
| HF | AABR07051450.1 | protein coding | 853.1037648 | 549.706917 | 1.55 | 1.26 | 1.91 | 0.00 | 0.21 | 3 | 15912485 | 15923042 | 10557 | + |
| HF | Pilrb2l2 | protein coding | 21.95426772 | 0.179222828 | 122.50 | 11.79 | 1272.21 | 0.00 | 0.23 | 12 | 18210535 | 18582203 | 371668 | - |
| HF | ENSRNOG00000066099 | lncRNA | 4.176285287 | 17.60979917 | -4.22 | -9.21 | -1.93 | 0.00 | 0.99 | 1 | 53816738 | 53818754 | 2016 | - |
| HF | LOC120100310 | snoRNA | 8.159795907 | 1.365377638 | 5.98 | 2.16 | 16.52 | 0.00 | 1.00 | 1 | 110972130 | 110972223 | 93 | - |
| HF | Glt8d2 | protein coding | 14.02146449 | 37.30411155 | -2.66 | -4.72 | -1.50 | 0.00 | 1.00 | 7 | 21029525 | 21081081 | 51556 | + |
| HF | Adam1b | protein coding | 11.68084766 | 2.586426264 | 4.52 | 1.85 | 11.01 | 0.00 | 1.00 | 12 | 35037343 | 35041083 | 3740 | + |
| HF | Dpm2 | protein coding | 18.26631649 | 52.21349101 | -2.86 | -5.46 | -1.50 | 0.00 | 1.00 | 3 | 15856182 | 15869166 | 12984 | + |
| HF | Cd6 | protein coding | 4.776125175 | 12.87044281 | -2.69 | -4.97 | -1.46 | 0.00 | 1.00 | 1 | 207442877 | 207481635 | 38758 | - |
| HF | Mov10l1 | protein coding | 1.5002152 | 7.031999232 | -4.69 | -12.53 | -1.75 | 0.00 | 1.00 | 7 | 120070135 | 120134766 | 64631 | + |
| HF | Steap4 | protein coding | 6.544293429 | 1.906610674 | 3.43 | 1.56 | 7.57 | 0.00 | 1.00 | 4 | 26054671 | 26099860 | 45189 | - |
| HF | Col14a1 | protein coding | 1.758951726 | 7.193091472 | -4.09 | -10.16 | -1.65 | 0.00 | 1.00 | 7 | 86722094 | 86937215 | 215121 | + |
| HF | Myo3b | protein coding | 5.518180285 | 1.235955002 | 4.46 | 1.69 | 11.82 | 0.00 | 1.00 | 3 | 54820251 | 55227374 | 407123 | + |
| HF | Il18 | protein coding | 13.70313298 | 26.45866112 | -1.93 | -2.96 | -1.26 | 0.00 | 1.00 | 8 | 50906960 | 50932888 | 25928 | + |
| HF | Cntf | protein coding | 38.80668076 | 23.58869855 | 1.65 | 1.19 | 2.28 | 0.00 | 1.00 | 1 | 209887854 | 209889878 | 2024 | - |
| HF | Vom2r44 | protein coding | 23.72240351 | 52.78763781 | -2.23 | -3.76 | -1.32 | 0.00 | 1.00 | 2 | 148500170 | 148531881 | 31711 | + |
| HF | Tacr3 | protein coding | 8.459550505 | 38.08639925 | -4.50 | -12.12 | -1.67 | 0.00 | 1.00 | 2 | 223266536 | 223363792 | 97256 | + |
| HF | LOC108348276 | protein coding | 61.04980034 | 36.95003899 | 1.65 | 1.18 | 2.31 | 0.00 | 1.00 | 2 | 29612925 | 29623731 | 10806 | + |
| HF | Hscb | protein coding | 24.808953 | 40.37102904 | -1.63 | -2.25 | -1.18 | 0.00 | 1.00 | 12 | 45821555 | 45831910 | 10355 | + |
| HF | ENSRNOG00000066012 | lncRNA | 1215.250608 | 795.4119423 | 1.53 | 1.15 | 2.03 | 0.00 | 1.00 | 19 | 13736990 | 13760944 | 23954 | - |
| HF | Npr3 | protein coding | 5.603740435 | 19.64808794 | -3.51 | -8.13 | -1.51 | 0.00 | 1.00 | 2 | 60870594 | 60932956 | 62362 | - |
| HF | Slc24a1 | protein coding | 1.256699571 | 5.221782222 | -4.16 | -11.21 | -1.54 | 0.00 | 1.00 | 8 | 65440730 | 65466002 | 25272 | - |
| HF | Fscn3 | protein coding | 0.305027033 | 2.879690011 | -9.44 | -45.54 | -1.96 | 0.01 | 1.00 | 4 | 57042797 | 57052405 | 9608 | + |
| HF | Ggn | protein coding | 1.255100748 | 5.291656572 | -4.22 | -11.61 | -1.53 | 0.01 | 1.00 | 1 | 84470508 | 84474115 | 3607 | + |
| HF | ENSRNOG00000067940 | lncRNA | 3.505068779 | 9.991956171 | -2.85 | -6.00 | -1.35 | 0.01 | 1.00 | 2 | 182593209 | 182598659 | 5450 | + |
| HF | Csf3r | protein coding | 20.0585444 | 32.10243299 | -1.60 | -2.25 | -1.14 | 0.01 | 1.00 | 5 | 138301506 | 138317882 | 16376 | + |
| HF | Lilrb3 | protein coding | 1.004967208 | 5.247980521 | -5.22 | -17.26 | -1.58 | 0.01 | 1.00 | 1 | 69928945 | 69939049 | 10104 | - |
| HF | LOC100362400 | protein coding | 10.75373662 | 4.854914441 | 2.22 | 1.23 | 3.99 | 0.01 | 1.00 | 8 | 5697563 | 5698306 | 743 | + |
| HF | Tshz2 | protein coding | 248.0629673 | 594.329679 | -2.40 | -4.60 | -1.25 | 0.01 | 1.00 | 3 | 158405423 | 158850129 | 444706 | + |
| HF | Vwc2l | protein coding | 12.5055524 | 24.42294032 | -1.95 | -3.23 | -1.18 | 0.01 | 1.00 | 9 | 72253387 | 72444840 | 191453 | + |
| HF | Egfl8 | protein coding | 20.25320362 | 11.99385345 | 1.69 | 1.14 | 2.50 | 0.01 | 1.00 | 20 | 4133629 | 4136019 | 2390 | + |
| HF | Zfp663 | protein coding | 1.259629155 | 4.853699698 | -3.85 | -10.63 | -1.40 | 0.01 | 1.00 | 3 | 154085957 | 154093161 | 7204 | - |
| HF | Igflr1 | protein coding | 7.730323648 | 15.92309139 | -2.06 | -3.57 | -1.19 | 0.01 | 1.00 | 1 | 85816326 | 85821031 | 4705 | + |
| PFC | Pilrb2l2 | protein coding | 26.22849279 | 0.189204593 | 138.63 | 17.55 | 1095.16 | 0.00 | 0.05 | 12 | 18210535 | 18582203 | 371668 | - |
| PFC | ENSRNOG00000066099 | lncRNA | 5.746373925 | 22.67754149 | -3.95 | -7.27 | -2.14 | 0.00 | 0.09 | 1 | 53816738 | 53818754 | 2016 | - |
| PFC | ENSRNOG00000069549 | protein coding | 735.8740085 | 32.35144946 | 22.75 | 5.25 | 98.52 | 0.00 | 0.16 | 5 | 23768429 | 23768807 | 378 | + |
| PFC | LOC120103376 | snRNA | 17.08955216 | 4.416160537 | 3.87 | 2.00 | 7.50 | 0.00 | 0.21 | 5 | 130667018 | 130667134 | 116 | + |
| PFC | Vom2r44 | protein coding | 25.58860837 | 62.77407649 | -2.45 | -3.81 | -1.58 | 0.00 | 0.21 | 2 | 148500170 | 148531881 | 31711 | + |
| PFC | ENSRNOG00000069434 | protein coding | 12.47500501 | 29.78238178 | -2.39 | -3.74 | -1.52 | 0.00 | 0.29 | 1 | 196028296 | 196037444 | 9148 | - |
| PFC | Nat8f1 | protein coding | 24.16350187 | 45.68706435 | -1.89 | -2.70 | -1.33 | 0.00 | 0.50 | 4 | 118332862 | 118344036 | 11174 | - |
| PFC | Hmgb2 | protein coding | 8.126310714 | 0.606774825 | 13.39 | 3.08 | 58.27 | 0.00 | 0.52 | 16 | 32710658 | 32713141 | 2483 | - |
| PFC | Gstm4 | protein coding | 41.51565326 | 62.94052198 | -1.52 | -1.93 | -1.19 | 0.00 | 0.58 | 2 | 195680004 | 195685324 | 5320 | - |
| PFC | LOC120102105 | rRNA | 1316112.404 | 87938.37585 | 14.97 | 3.01 | 74.32 | 0.00 | 0.69 | 3 | 2231807 | 2233679 | 1872 | + |
| PFC | Arl11 | protein coding | 10.03593872 | 20.45083879 | -2.04 | -3.11 | -1.33 | 0.00 | 0.69 | 15 | 35480018 | 35496597 | 16579 | + |
| PFC | LOC120101310 | snRNA | 4541.820519 | 2283.311306 | 1.99 | 1.31 | 3.01 | 0.00 | 0.69 | 2 | 183901212 | 183901377 | 165 | + |
| PFC | LOC120103261 | snoRNA | 10.63554789 | 3.419938232 | 3.11 | 1.54 | 6.29 | 0.00 | 0.69 | 5 | 144505211 | 144505347 | 136 | + |
| PFC | Dnmt3b | protein coding | 7.655994811 | 15.12731952 | -1.98 | -3.04 | -1.28 | 0.00 | 0.69 | 3 | 142130592 | 142169125 | 38533 | + |
| PFC | ENSRNOG00000066166 | protein coding | 24.91440405 | 13.38196248 | 1.86 | 1.25 | 2.76 | 0.00 | 0.69 | 13 | 62124375 | 62124888 | 513 | + |
| PFC | Diras3 | protein coding | 11.63972644 | 21.58665783 | -1.85 | -2.75 | -1.25 | 0.00 | 0.69 | 6 | 130524742 | 130530826 | 6084 | - |
| PFC | Gsta1 | protein coding | 236.9483864 | 358.6257026 | -1.51 | -1.98 | -1.16 | 0.00 | 0.69 | 9 | 23703477 | 23720122 | 16645 | - |
| PFC | Kcnh6 | protein coding | 10.48825433 | 21.41291621 | -2.04 | -3.26 | -1.28 | 0.00 | 0.69 | 10 | 90949846 | 90970702 | 20856 | + |
| PFC | Hist1h2bc | protein coding | 24.62483104 | 13.65795694 | 1.80 | 1.22 | 2.66 | 0.00 | 0.69 | 17 | 42694723 | 42696643 | 1920 | - |
| PFC | ENSRNOG00000063301 | protein coding | 1535.306951 | 197.3715923 | 7.78 | 2.01 | 30.09 | 0.00 | 0.69 | 3 | 2237964 | 2242883 | 4919 | - |
| PFC | AABR07011698.1 | protein coding | 15.82690917 | 7.979954717 | 1.98 | 1.26 | 3.12 | 0.00 | 0.69 | 2 | 153941154 | 153942720 | 1566 | - |
| PFC | AABR07030375.1 | protein coding | 49.6776959 | 25.47833503 | 1.95 | 1.25 | 3.05 | 0.00 | 0.69 | 10 | 80274041 | 80274497 | 456 | - |
| PFC | LOC120100310 | snoRNA | 11.2648962 | 3.577766085 | 3.15 | 1.46 | 6.79 | 0.00 | 0.69 | 1 | 110972130 | 110972223 | 93 | - |
| PFC | LOC679731 | pseudogene | 99.51279136 | 62.26531899 | 1.60 | 1.17 | 2.19 | 0.00 | 0.69 | 1 | 39601959 | 39603123 | 1164 | - |
| PFC | Myo3a | protein coding | 19.59966025 | 9.63973222 | 2.03 | 1.26 | 3.28 | 0.00 | 0.69 | 17 | 84543552 | 84759043 | 215491 | + |
| PFC | Kcnq4 | protein coding | 27.06993602 | 15.14746587 | 1.79 | 1.21 | 2.65 | 0.00 | 0.69 | 5 | 134275934 | 134326933 | 50999 | - |
| PFC | Ifi35 | protein coding | 14.30195395 | 24.07886105 | -1.68 | -2.40 | -1.18 | 0.00 | 0.69 | 10 | 86381010 | 86389953 | 8943 | + |
| PFC | Adamts18 | protein coding | 11.6350008 | 22.48976257 | -1.93 | -3.03 | -1.23 | 0.00 | 0.69 | 19 | 41688617 | 41839782 | 151165 | - |
| PFC | LOC120094550 | snRNA | 30.97117464 | 11.9455309 | 2.59 | 1.36 | 4.96 | 0.00 | 0.69 | 8 | 65717512 | 65717628 | 116 | - |
| PFC | Omp | protein coding | 22.87126316 | 44.52062875 | -1.95 | -3.07 | -1.24 | 0.00 | 0.69 | 1 | 152433652 | 152441923 | 8271 | - |
| PFC | Abcg3 | protein coding | 3.57908165 | 0.200880625 | 17.82 | 2.50 | 127.09 | 0.00 | 0.69 | 14 | 4730453 | 4839245 | 108792 | + |
| PFC | LOC120101254 | scaRNA | 39.00306765 | 22.21321262 | 1.76 | 1.19 | 2.58 | 0.00 | 0.69 | 2 | 174156951 | 174157079 | 128 | + |
| PFC | LOC120094045 | snRNA | 58.15353532 | 29.74618932 | 1.95 | 1.23 | 3.10 | 0.00 | 0.69 | 7 | 13423040 | 13423156 | 116 | + |
| PFC | Ggt6 | protein coding | 18.48950881 | 33.80570639 | -1.83 | -2.77 | -1.21 | 0.00 | 0.69 | 10 | 57051825 | 57067261 | 15436 | + |
| PFC | Sp7 | protein coding | 21.21417424 | 40.41936381 | -1.91 | -2.98 | -1.22 | 0.00 | 0.72 | 7 | 133484609 | 133494848 | 10239 | - |
| PFC | Rnu1-136 | snRNA | 460.7047816 | 242.414079 | 1.90 | 1.22 | 2.97 | 0.00 | 0.72 | 3 | 12149416 | 12149580 | 164 | - |
| PFC | LOC103693194 | lncRNA | 10.27253402 | 18.37543786 | -1.79 | -2.69 | -1.19 | 0.01 | 0.72 | 9 | 1046547 | 1049875 | 3328 | + |
| PFC | Slc16a11 | protein coding | 88.11818496 | 143.2211096 | -1.63 | -2.29 | -1.15 | 0.01 | 0.72 | 10 | 54927725 | 54942916 | 15191 | + |
| PFC | Il12rb2 | protein coding | 7.062191989 | 2.28387079 | 3.09 | 1.39 | 6.86 | 0.01 | 0.72 | 4 | 96426842 | 96515290 | 88448 | - |
| PFC | ENSRNOG00000066836 | protein coding | 52.21736536 | 79.71890383 | -1.53 | -2.06 | -1.13 | 0.01 | 0.72 | 8 | 3228866 | 3234904 | 6038 | - |
| PFC | ENSRNOG00000063145 | lncRNA | 105.2472869 | 65.94670375 | 1.60 | 1.15 | 2.22 | 0.01 | 0.72 | 1 | 114719526 | 114753075 | 33549 | - |
| PFC | Nme3 | protein coding | 35.78070038 | 19.59236133 | 1.83 | 1.19 | 2.80 | 0.01 | 0.73 | 10 | 13917403 | 13918360 | 957 | + |
| PFC | Acot4 | protein coding | 20.21511247 | 33.79817673 | -1.67 | -2.42 | -1.16 | 0.01 | 0.73 | 6 | 103668753 | 103673918 | 5165 | + |
| PFC | Clspn | protein coding | 34.68611577 | 19.98800765 | 1.74 | 1.17 | 2.58 | 0.01 | 0.73 | 5 | 138850128 | 138885035 | 34907 | + |
| PFC | LOC685311 | protein coding | 8.18886759 | 15.11636586 | -1.85 | -2.88 | -1.18 | 0.01 | 0.74 | 5 | 68634989 | 68635358 | 369 | - |
| PFC | ENSRNOG00000069208 | pseudogene | 88.36028127 | 24.07708866 | 3.67 | 1.43 | 9.41 | 0.01 | 0.74 | 3 | 2233522 | 2234360 | 838 | - |
| PFC | LOC120095385 | snRNA | 7171.503663 | 4226.7772 | 1.70 | 1.15 | 2.50 | 0.01 | 0.74 | 10 | 72249871 | 72250035 | 164 | + |
| PFC | AC080157.1 | protein coding | 58.80471712 | 127.3895814 | -2.17 | -3.82 | -1.23 | 0.01 | 0.74 | 1 | 113280886 | 113287666 | 6780 | - |
| PFC | Snora49 | snoRNA | 35.53476875 | 15.89838358 | 2.24 | 1.24 | 4.04 | 0.01 | 0.74 | 12 | 45970884 | 45971017 | 133 | + |
| PFC | Prickle3 | protein coding | 10.12913236 | 16.74161383 | -1.65 | -2.39 | -1.14 | 0.01 | 0.74 | X | 14837650 | 14848219 | 10569 | - |
| PFC | LOC108351472 | lncRNA | 6.92907838 | 12.901323 | -1.86 | -2.96 | -1.17 | 0.01 | 0.75 | 7 | 62800401 | 62804992 | 4591 | - |
| PFC | Eln | protein coding | 59.96025149 | 39.57980924 | 1.51 | 1.11 | 2.07 | 0.01 | 0.75 | 12 | 21968544 | 22011929 | 43385 | + |
| PFC | Twist1 | protein coding | 1.666967108 | 4.67172895 | -2.80 | -6.08 | -1.29 | 0.01 | 0.75 | 6 | 50674678 | 50677654 | 2976 | + |
| PFC | Mcm5 | protein coding | 17.54758825 | 9.574393546 | 1.83 | 1.16 | 2.89 | 0.01 | 0.75 | 19 | 13483066 | 13504390 | 21324 | + |
| PFC | ENSRNOG00000063880 | pseudogene | 76.6163719 | 116.7642001 | -1.52 | -2.09 | -1.11 | 0.01 | 0.75 | 20 | 7889554 | 7889990 | 436 | - |
| PFC | Cps1 | protein coding | 7.915697299 | 3.503231387 | 2.26 | 1.22 | 4.18 | 0.01 | 0.75 | 9 | 68614153 | 68737034 | 122881 | + |
| PFC | ENSRNOG00000069912 | protein coding | 0.185014934 | 4.986037991 | -26.95 | -323.67 | -2.24 | 0.01 | 0.75 | 11 | 56713182 | 56718571 | 5389 | + |
| PFC | Cntf | protein coding | 16.07975807 | 24.3872216 | -1.52 | -2.08 | -1.11 | 0.01 | 0.75 | 1 | 209887854 | 209889878 | 2024 | - |
| PFC | Ebf2 | protein coding | 1.580433224 | 5.373043001 | -3.40 | -8.60 | -1.34 | 0.01 | 0.75 | 15 | 41435509 | 41634404 | 198895 | + |
| 1. Nucleus accumbens is ACB, hippocampus is HF, and prefrontal cortex is PFC. | | | | | | | | | | | | | | |
| 2. Least Squares mean refers to the average expression level for a gene. | | | | | | | | | | | | | | |
| 3. Cells with false discovery rate (FDR)-adjusted p value <0.05 were highlighted. | | | | | | | | | | | | | | |

| **Supplemental Table 2.** Pathway enrichment results based on differential gene expression results due to PFOS exposure in rats using Kyoto Encyclopedia of Genes and Genomes (KEGG). | | | | | |
| --- | --- | --- | --- | --- | --- |
| **Tissue Type^1^** | **Pathway** | **Enrichment Score^2^** | **P Value** | **FDR-Adjsuted P Value^3^** | **Genes** |
| ACB | ECM-receptor interaction | 16.21 | 0.00 | 0.00 | COL1A1/COL1A2/COL4A5/COL4A6/COL6A3/ITGB4 |
| ACB | Protein digestion and absorption | 15.30 | 0.00 | 0.00 | COL1A1/COL1A2/COL4A5/COL4A6/COL6A3/SLC9A3 |
| ACB | Focal adhesion | 11.38 | 0.00 | 0.00 | COL1A1/COL1A2/COL4A5/COL4A6/COL6A3/ITGB4 |
| ACB | PI3K-Akt signaling pathway | 10.85 | 0.00 | 0.00 | COL1A1/COL1A2/COL4A5/COL4A6/COL6A3/ITGB4/MYC |
| ACB | Amoebiasis | 8.94 | 0.00 | 0.01 | COL1A1/COL1A2/COL4A5/COL4A6 |
| ACB | AGE-RAGE signaling pathway in diabetic complications | 8.86 | 0.00 | 0.01 | COL1A1/COL1A2/COL4A5/COL4A6 |
| ACB | Human papillomavirus infection | 8.53 | 0.00 | 0.01 | COL1A1/COL1A2/COL4A5/COL4A6/COL6A3/ITGB4 |
| ACB | Relaxin signaling pathway | 7.99 | 0.00 | 0.01 | COL1A1/COL1A2/COL4A5/COL4A6 |
| ACB | Small cell lung cancer | 6.24 | 0.00 | 0.07 | COL4A5/COL4A6/MYC |
| ACB | TGF-beta signaling pathway | 6.12 | 0.00 | 0.08 | BMP7/MYC/THSD4 |
| ACB | Thyroid cancer | 5.40 | 0.00 | 0.13 | CDH1/MYC |
| ACB | Platelet activation | 5.38 | 0.00 | 0.13 | BTK/COL1A1/COL1A2 |
| ACB | Bladder cancer | 5.24 | 0.01 | 0.14 | CDH1/MYC |
| ACB | Hippo signaling pathway | 4.73 | 0.01 | 0.22 | BMP7/CDH1/MYC |
| ACB | Endometrial cancer | 4.51 | 0.01 | 0.25 | CDH1/MYC |
| ACB | Epstein-Barr virus infection | 4.02 | 0.02 | 0.37 | BTK/MYC/VIM |
| ACB | Proteoglycans in cancer | 4.00 | 0.02 | 0.37 | COL1A1/COL1A2/MYC |
| ACB | Bile secretion | 3.79 | 0.02 | 0.43 | SLC22A8/SLC9A3 |
| ACB | Pathways in cancer | 2.95 | 0.05 | 0.94 | CDH1/COL4A5/COL4A6/MYC |
| ACB | Taurine and hypotaurine metabolism | 2.90 | 0.06 | 0.94 | FMO1 |
| ACB | Proximal tubule bicarbonate reclamation | 2.85 | 0.06 | 0.94 | SLC9A3 |
| ACB | Gastric cancer | 2.74 | 0.06 | 1.00 | CDH1/MYC |
| ACB | Primary immunodeficiency | 2.41 | 0.09 | 1.00 | BTK |
| ACB | Diabetic cardiomyopathy | 2.36 | 0.09 | 1.00 | COL1A1/COL1A2 |
| ACB | Drug metabolism - cytochrome P450 | 1.95 | 0.14 | 1.00 | FMO1 |
| ACB | Mineral absorption | 1.95 | 0.14 | 1.00 | SLC9A3 |
| ACB | Salmonella infection | 1.92 | 0.15 | 1.00 | ANXA2/MYC |
| ACB | GnRH secretion | 1.82 | 0.16 | 1.00 | KISS1R |
| ACB | Glutathione metabolism | 1.81 | 0.16 | 1.00 | NAT8F2 |
| ACB | Fc epsilon RI signaling pathway | 1.79 | 0.17 | 1.00 | BTK |
| ACB | Central carbon metabolism in cancer | 1.78 | 0.17 | 1.00 | MYC |
| ACB | Acute myeloid leukemia | 1.77 | 0.17 | 1.00 | MYC |
| ACB | MicroRNAs in cancer | 1.76 | 0.17 | 1.00 | MYC/VIM |
| ACB | Adherens junction | 1.72 | 0.18 | 1.00 | CDH1 |
| ACB | Retinol metabolism | 1.71 | 0.18 | 1.00 | ALDH1A3 |
| ACB | Melanoma | 1.70 | 0.18 | 1.00 | CDH1 |
| ACB | Gastric acid secretion | 1.67 | 0.19 | 1.00 | SLC26A7 |
| ACB | B cell receptor signaling pathway | 1.66 | 0.19 | 1.00 | BTK |
| ACB | Arrhythmogenic right ventricular cardiomyopathy | 1.65 | 0.19 | 1.00 | ITGB4 |
| ACB | Bacterial invasion of epithelial cells | 1.64 | 0.19 | 1.00 | CDH1 |
| ACB | Chronic myeloid leukemia | 1.64 | 0.19 | 1.00 | MYC |
| ACB | ErbB signaling pathway | 1.56 | 0.21 | 1.00 | MYC |
| ACB | Colorectal cancer | 1.54 | 0.22 | 1.00 | MYC |
| ACB | Hypertrophic cardiomyopathy | 1.49 | 0.23 | 1.00 | ITGB4 |
| ACB | Dilated cardiomyopathy | 1.47 | 0.23 | 1.00 | ITGB4 |
| ACB | NF-kappa B signaling pathway | 1.43 | 0.24 | 1.00 | BTK |
| ACB | Osteoclast differentiation | 1.26 | 0.28 | 1.00 | BTK |
| ACB | Thyroid hormone signaling pathway | 1.25 | 0.29 | 1.00 | MYC |
| ACB | Cell cycle | 1.22 | 0.30 | 1.00 | MYC |
| ACB | Apelin signaling pathway | 1.16 | 0.31 | 1.00 | CDH1 |
| ACB | Signaling pathways regulating pluripotency of stem cells | 1.13 | 0.32 | 1.00 | MYC |
| ACB | Hepatitis C | 1.10 | 0.33 | 1.00 | MYC |
| ACB | JAK-STAT signaling pathway | 1.08 | 0.34 | 1.00 | MYC |
| ACB | Hepatitis B | 1.08 | 0.34 | 1.00 | MYC |
| ACB | Breast cancer | 1.07 | 0.34 | 1.00 | MYC |
| ACB | Cell adhesion molecules | 1.01 | 0.37 | 1.00 | CDH1 |
| ACB | Cellular senescence | 0.98 | 0.38 | 1.00 | MYC |
| ACB | Wnt signaling pathway | 0.97 | 0.38 | 1.00 | MYC |
| ACB | Hepatocellular carcinoma | 0.96 | 0.38 | 1.00 | MYC |
| ACB | Axon guidance | 0.92 | 0.40 | 1.00 | BMP7 |
| ACB | Transcriptional misregulation in cancer | 0.90 | 0.41 | 1.00 | MYC |
| ACB | Kaposi sarcoma-associated herpesvirus infection | 0.88 | 0.42 | 1.00 | MYC |
| ACB | Chemical carcinogenesis - receptor activation | 0.81 | 0.45 | 1.00 | MYC |
| ACB | Rap1 signaling pathway | 0.80 | 0.45 | 1.00 | CDH1 |
| ACB | Regulation of actin cytoskeleton | 0.78 | 0.46 | 1.00 | ITGB4 |
| ACB | Human cytomegalovirus infection | 0.75 | 0.47 | 1.00 | MYC |
| ACB | Human T-cell leukemia virus 1 infection | 0.74 | 0.48 | 1.00 | MYC |
| ACB | Cytokine-cytokine receptor interaction | 0.64 | 0.52 | 1.00 | BMP7 |
| ACB | MAPK signaling pathway | 0.57 | 0.56 | 1.00 | MYC |
| ACB | Neuroactive ligand-receptor interaction | 0.44 | 0.65 | 1.00 | KISS1R |
| ACB | Metabolic pathways | 0.20 | 0.82 | 1.00 | ALDH1A3/FMO1/NAT8F2 |
| HF | Cytokine-cytokine receptor interaction | 5.35 | 0.00 | 1.00 | CNTF/CSF3R/IL18 |
| HF | JAK-STAT signaling pathway | 4.08 | 0.02 | 1.00 | CNTF/CSF3R |
| HF | Glycosylphosphatidylinositol (GPI)-anchor biosynthesis | 3.40 | 0.03 | 1.00 | DPM2 |
| HF | Phototransduction | 3.36 | 0.03 | 1.00 | SLC24A1 |
| HF | African trypanosomiasis | 3.03 | 0.05 | 1.00 | IL18 |
| HF | Cytosolic DNA-sensing pathway | 2.74 | 0.06 | 1.00 | IL18 |
| HF | Malaria | 2.72 | 0.07 | 1.00 | IL18 |
| HF | N-Glycan biosynthesis | 2.70 | 0.07 | 1.00 | DPM2 |
| HF | Legionellosis | 2.61 | 0.07 | 1.00 | IL18 |
| HF | Inflammatory bowel disease | 2.52 | 0.08 | 1.00 | IL18 |
| HF | B cell receptor signaling pathway | 2.30 | 0.10 | 1.00 | LILRB3 |
| HF | Viral protein interaction with cytokine and cytokine receptor | 2.19 | 0.11 | 1.00 | IL18 |
| HF | Rheumatoid arthritis | 2.18 | 0.11 | 1.00 | IL18 |
| HF | Hematopoietic cell lineage | 2.17 | 0.11 | 1.00 | CSF3R |
| HF | Protein digestion and absorption | 2.02 | 0.13 | 1.00 | COL14A1 |
| HF | Osteoclast differentiation | 1.87 | 0.15 | 1.00 | LILRB3 |
| HF | Yersinia infection | 1.77 | 0.17 | 1.00 | IL18 |
| HF | Influenza A | 1.63 | 0.20 | 1.00 | IL18 |
| HF | Cell adhesion molecules | 1.59 | 0.20 | 1.00 | CD6 |
| HF | Tuberculosis | 1.58 | 0.21 | 1.00 | IL18 |
| HF | NOD-like receptor signaling pathway | 1.56 | 0.21 | 1.00 | IL18 |
| HF | Lipid and atherosclerosis | 1.41 | 0.24 | 1.00 | IL18 |
| HF | Calcium signaling pathway | 1.25 | 0.29 | 1.00 | TACR3 |
| HF | Salmonella infection | 1.23 | 0.29 | 1.00 | IL18 |
| HF | Herpes simplex virus 1 infection | 1.07 | 0.34 | 1.00 | ZFP663 |
| HF | PI3K-Akt signaling pathway | 0.97 | 0.38 | 1.00 | CSF3R |
| HF | Neuroactive ligand-receptor interaction | 0.90 | 0.41 | 1.00 | TACR3 |
| HF | Pathways in cancer | 0.64 | 0.53 | 1.00 | CSF3R |
| HF | Metabolic pathways | 0.10 | 0.91 | 1.00 | DPM2 |
| PFC | Glutathione metabolism | 11.55 | 0.00 | 0.00 | GGT6/GSTA1/GSTM4/NAT8F1 |
| PFC | Drug metabolism - other enzymes | 7.45 | 0.00 | 0.10 | GSTA1/GSTM4/NME3 |
| PFC | Metabolism of xenobiotics by cytochrome P450 | 5.09 | 0.01 | 0.49 | GSTA1/GSTM4 |
| PFC | Drug metabolism - cytochrome P450 | 5.05 | 0.01 | 0.49 | GSTA1/GSTM4 |
| PFC | Chemical carcinogenesis - DNA adducts | 4.94 | 0.01 | 0.49 | GSTA1/GSTM4 |
| PFC | Metabolic pathways | 4.67 | 0.01 | 0.54 | ACOT4/CPS1/DNMT3B/GGT6/GSTA1/GSTM4/NAT8F1/NME3 |
| PFC | Platinum drug resistance | 4.39 | 0.01 | 0.61 | GSTA1/GSTM4 |
| PFC | Nitrogen metabolism | 4.15 | 0.02 | 0.67 | CPS1 |
| PFC | Fluid shear stress and atherosclerosis | 3.25 | 0.04 | 1.00 | GSTA1/GSTM4 |
| PFC | JAK-STAT signaling pathway | 3.18 | 0.04 | 1.00 | CNTF/IL12RB2 |
| PFC | Arginine biosynthesis | 3.17 | 0.04 | 1.00 | CPS1 |
| PFC | Taurine and hypotaurine metabolism | 3.12 | 0.04 | 1.00 | GGT6 |
| PFC | Hepatocellular carcinoma | 2.91 | 0.05 | 1.00 | GSTA1/GSTM4 |
| PFC | Fatty acid elongation | 2.79 | 0.06 | 1.00 | ACOT4 |
| PFC | Biosynthesis of unsaturated fatty acids | 2.66 | 0.07 | 1.00 | ACOT4 |
| PFC | Alanine, aspartate and glutamate metabolism | 2.60 | 0.07 | 1.00 | CPS1 |
| PFC | DNA replication | 2.60 | 0.07 | 1.00 | MCM5 |
| PFC | Chemical carcinogenesis - reactive oxygen species | 2.59 | 0.07 | 1.00 | GSTA1/GSTM4 |
| PFC | Chemical carcinogenesis - receptor activation | 2.57 | 0.08 | 1.00 | GSTA1/GSTM4 |
| PFC | Pathways in cancer | 2.25 | 0.10 | 1.00 | GSTA1/GSTM4/IL12RB2 |
| PFC | Cysteine and methionine metabolism | 2.23 | 0.11 | 1.00 | DNMT3B |
| PFC | Pyrimidine metabolism | 2.18 | 0.11 | 1.00 | NME3 |
| PFC | Cytokine-cytokine receptor interaction | 2.17 | 0.11 | 1.00 | CNTF/IL12RB2 |
| PFC | Ovarian steroidogenesis | 2.12 | 0.12 | 1.00 | ACOT4 |
| PFC | Inflammatory bowel disease | 2.08 | 0.13 | 1.00 | IL12RB2 |
| PFC | Biosynthesis of amino acids | 1.90 | 0.15 | 1.00 | CPS1 |
| PFC | Nucleotide metabolism | 1.84 | 0.16 | 1.00 | NME3 |
| PFC | Systemic lupus erythematosus | 1.80 | 0.16 | 1.00 | HIST1H2BC |
| PFC | Th1 and Th2 cell differentiation | 1.74 | 0.18 | 1.00 | IL12RB2 |
| PFC | Protein digestion and absorption | 1.59 | 0.20 | 1.00 | ELN |
| PFC | Cholinergic synapse | 1.53 | 0.22 | 1.00 | KCNQ4 |
| PFC | Carbon metabolism | 1.50 | 0.22 | 1.00 | CPS1 |
| PFC | Purine metabolism | 1.41 | 0.24 | 1.00 | NME3 |
| PFC | Cell cycle | 1.41 | 0.24 | 1.00 | MCM5 |
| PFC | Neutrophil extracellular trap formation | 1.35 | 0.26 | 1.00 | HIST1H2BC |
| PFC | Alcoholism | 1.34 | 0.26 | 1.00 | HIST1H2BC |
| PFC | Biosynthesis of cofactors | 1.30 | 0.27 | 1.00 | NME3 |
| PFC | Wnt signaling pathway | 1.15 | 0.32 | 1.00 | PRICKLE3 |
| PFC | Viral carcinogenesis | 1.05 | 0.35 | 1.00 | HIST1H2BC |
| PFC | Proteoglycans in cancer | 0.99 | 0.37 | 1.00 | TWIST1 |
| PFC | MicroRNAs in cancer | 0.78 | 0.46 | 1.00 | DNMT3B |
| 1. Nucleus accumbens is ACB, hippocampus is HF, and prefrontal cortex is PFC. | | | | | |
| 2. An enrichment score quantifies how much a set of genes (representing a pathway or function) is over-represented at the top or bottom of a ranked list of genes, indicating enrichment or depletion of that pathway. | | | | | |
| 3. Cells with false discovery rate (FDR)-adjusted p value <0.05 were highlighted. | | | | | |

| **Table S3.** Summary statistical of t tests comparing PFOS-exposed and vehicle groups. | | | | | | | |
| --- | --- | --- | --- | --- | --- | --- | --- |
| **Metric^1^** | **Mean (PFOS)** | **Mean (Vehicle)** | **Difference (PFOS − Vehicle)** | **95% CI** | **t statistic** | **df** | **p-value** |
| Set Shifting | 270.75 | 173.5 | 97.25 | –117.26 to 311.76 | –0.976 | 13.51 | 0.346 |
| CHL (AUC) | 10.53 | 19.95 | –9.42 | –19.11 to 0.26 | 2.194 | 9.23 | 0.055 |
| Omissions (AUC) | 5.09 | 0.81 | 4.28 | 0.50 to 8.07 | –2.633 | 7.6 | 0.031 |
| Latency (AUC) | 86.64 | 69.2 | 17.44 | –27.39 to 62.28 | –0.850 | 11.77 | 0.412 |
| 1. Area under the curve (AUC) for percent preference for the large reward (CHL), response omissions (Omit), and response latency (Latency). For %CHL, a higher AUC reflects better delay tolerance (less impulsivity), whereas, for % omissions and response latency, a higher AUC reflects poorer neurobehavioral performance (greater omissions or slower responding). | | | | | | | |

| **Table S4.** Pathway enrichment results based on differential gene expression results for each of the neurobehavioral tests in rats using Kyoto Encyclopedia of Genes and Genomes (KEGG). | | | | |
| --- | --- | --- | --- | --- |
| **Tissue Type^1^** | **Tests^2^** | **Pathway** | **Enrichment Score^3^** | **FDR-Adjusted P Value^4^** |
| ACB | CHL | Ribosome | 0.593695 | 2.84E-10 |
| ACB | CHL | Coronavirus disease - COVID-19 | 0.5382599 | 3.47E-09 |
| ACB | CHL | ECM-receptor interaction | -0.4587796 | 4.20E-07 |
| ACB | CHL | Systemic lupus erythematosus | 0.6017547 | 1.64E-04 |
| ACB | CHL | Staphylococcus aureus infection | 0.6203946 | 4.40E-03 |
| ACB | CHL | Focal adhesion | -0.2443482 | 7.21E-03 |
| ACB | CHL | Cytoskeleton in muscle cells | -0.2391316 | 8.00E-03 |
| ACB | CHL | Fanconi anemia pathway | 0.5673732 | 1.13E-02 |
| ACB | CHL | Chemical carcinogenesis - DNA adducts | 0.6282469 | 1.55E-02 |
| ACB | CHL | Hematopoietic cell lineage | 0.5543147 | 1.84E-02 |
| ACB | CHL | Carbohydrate digestion and absorption | 0.5887923 | 2.89E-02 |
| ACB | CHL | Starch and sucrose metabolism | 0.6204715 | 3.98E-02 |
| ACB | CHL | Intestinal immune network for IgA production | 0.6377003 | 4.51E-02 |
| HF | CHL | Calcium signaling pathway | -0.4477729 | 2.24E-05 |
| HF | CHL | Cytoskeleton in muscle cells | -0.4149264 | 4.45E-03 |
| HF | CHL | ECM-receptor interaction | -0.5032374 | 5.69E-03 |
| HF | CHL | Staphylococcus aureus infection | 0.6123164 | 1.46E-02 |
| HF | CHL | MAPK signaling pathway | -0.3630928 | 1.57E-02 |
| HF | CHL | Axon guidance | -0.396206 | 1.91E-02 |
| PFC | CHL | Ribosome | 0.5013928 | 6.04E-06 |
| PFC | CHL | Pathways in cancer | -0.372926 | 2.39E-03 |
| PFC | CHL | Motor proteins | -0.4372508 | 1.59E-02 |
| PFC | CHL | cAMP signaling pathway | -0.4240598 | 1.59E-02 |
| PFC | CHL | Retinol metabolism | 0.690057 | 1.63E-02 |
| PFC | CHL | Coronavirus disease - COVID-19 | 0.3545053 | 4.04E-02 |
| PFC | CHL | MicroRNAs in cancer | -0.4207225 | 4.96E-02 |
| ACB | Latency | Coronavirus disease - COVID-19 | 0.5325724 | 2.06E-09 |
| ACB | Latency | Ribosome | 0.5396213 | 3.13E-07 |
| ACB | Latency | Hematopoietic cell lineage | 0.6167623 | 7.53E-04 |
| ACB | Latency | Graft-versus-host disease | 0.6847059 | 1.50E-03 |
| ACB | Latency | Systemic lupus erythematosus | 0.5640766 | 1.55E-03 |
| ACB | Latency | Rheumatoid arthritis | 0.5745529 | 1.66E-03 |
| ACB | Latency | Type I diabetes mellitus | 0.6308773 | 2.12E-03 |
| ACB | Latency | Autoimmune thyroid disease | 0.6617531 | 2.67E-03 |
| ACB | Latency | Allograft rejection | 0.6576882 | 3.18E-03 |
| ACB | Latency | Retinol metabolism | 0.6566537 | 4.01E-03 |
| ACB | Latency | Intestinal immune network for IgA production | 0.6919237 | 4.56E-03 |
| ACB | Latency | Staphylococcus aureus infection | 0.6064804 | 4.98E-03 |
| ACB | Latency | Leishmaniasis | 0.5257663 | 1.99E-02 |
| ACB | Latency | Chemical carcinogenesis - DNA adducts | 0.6078596 | 2.13E-02 |
| ACB | Latency | Complement and coagulation cascades | 0.5457242 | 2.13E-02 |
| ACB | Latency | Protein digestion and absorption | 0.4924313 | 2.38E-02 |
| HF | Latency | Ribosome | 0.5769226 | 6.35E-07 |
| HF | Latency | Calcium signaling pathway | -0.3921926 | 1.67E-04 |
| HF | Latency | Axon guidance | -0.4164088 | 4.17E-04 |
| HF | Latency | Neuroactive ligand-receptor interaction | -0.3646689 | 6.61E-04 |
| HF | Latency | Coronavirus disease - COVID-19 | 0.4481453 | 2.94E-03 |
| HF | Latency | Oxytocin signaling pathway | -0.4134752 | 2.94E-03 |
| HF | Latency | Adrenergic signaling in cardiomyocytes | -0.4079138 | 5.14E-03 |
| HF | Latency | MAPK signaling pathway | -0.3291975 | 8.72E-03 |
| HF | Latency | Glutamatergic synapse | -0.4236994 | 8.97E-03 |
| HF | Latency | Circadian entrainment | -0.4282878 | 2.44E-02 |
| HF | Latency | Regulation of actin cytoskeleton | -0.3436825 | 2.44E-02 |
| HF | Latency | Thyroid hormone signaling pathway | -0.3943218 | 3.45E-02 |
| PFC | Latency | Ribosome | 0.5941978 | 1.98E-08 |
| PFC | Latency | Coronavirus disease - COVID-19 | 0.5199502 | 2.69E-06 |
| PFC | Latency | Oxidative phosphorylation | 0.542512 | 4.68E-04 |
| PFC | Latency | ECM-receptor interaction | -0.4180121 | 9.16E-03 |
| PFC | Latency | Cytoskeleton in muscle cells | -0.2989869 | 9.16E-03 |
| PFC | Latency | Taurine and hypotaurine metabolism | -0.7317129 | 1.60E-02 |
| PFC | Latency | Protein digestion and absorption | -0.4102986 | 2.62E-02 |
| PFC | Latency | Chemical carcinogenesis - reactive oxygen species | 0.4292925 | 2.62E-02 |
| PFC | Latency | Prion disease | 0.4164452 | 3.04E-02 |
| PFC | Latency | Thermogenesis | 0.4232188 | 3.42E-02 |
| PFC | Latency | Parkinson disease | 0.4106288 | 3.42E-02 |
| PFC | Latency | Huntington disease | 0.3965379 | 4.12E-02 |
| ACB | Omit | Neuroactive ligand-receptor interaction | 0.4389356 | 1.98E-05 |
| ACB | Omit | ECM-receptor interaction | 0.5020728 | 1.18E-02 |
| ACB | Omit | Olfactory transduction | 0.5352053 | 3.54E-02 |
| HF | Omit | ECM-receptor interaction | 0.6536968 | 1.80E-02 |
| PFC | Omit | Oxidative phosphorylation | 0.5752551 | 4.14E-05 |
| PFC | Omit | Parkinson disease | 0.4488108 | 2.77E-04 |
| PFC | Omit | Ribosome | 0.4904602 | 5.79E-04 |
| PFC | Omit | Prion disease | 0.4304044 | 5.79E-04 |
| PFC | Omit | Spliceosome | 0.4446546 | 8.30E-03 |
| PFC | Omit | Proteasome | 0.6387707 | 9.30E-03 |
| PFC | Omit | Coronavirus disease - COVID-19 | 0.4052376 | 1.39E-02 |
| PFC | Omit | Protein digestion and absorption | -0.5625386 | 1.61E-02 |
| PFC | Omit | Huntington disease | 0.3621436 | 1.82E-02 |
| PFC | Omit | Cell cycle | 0.414169 | 3.56E-02 |
| PFC | Omit | Non-alcoholic fatty liver disease | 0.4227272 | 3.67E-02 |
| ACB | SetShift | Neuroactive ligand-receptor interaction | -0.4346441 | 2.99E-08 |
| ACB | SetShift | Systemic lupus erythematosus | -0.4919854 | 1.27E-02 |
| ACB | SetShift | Hormone signaling | -0.3840385 | 1.27E-02 |
| ACB | SetShift | Chemical carcinogenesis - DNA adducts | -0.5628991 | 2.08E-02 |
| ACB | SetShift | Drug metabolism - cytochrome P450 | -0.5442988 | 2.08E-02 |
| ACB | SetShift | Metabolism of xenobiotics by cytochrome P450 | -0.5387185 | 2.08E-02 |
| ACB | SetShift | Glutathione metabolism | -0.4705054 | 2.23E-02 |
| ACB | SetShift | Drug metabolism - other enzymes | -0.4779368 | 4.43E-02 |
| HF | SetShift | Cytoskeleton in muscle cells | 0.4080842 | 1.26E-02 |
| PFC | SetShift | Ribosome | -0.6176924 | 1.07E-14 |
| PFC | SetShift | Coronavirus disease - COVID-19 | -0.3720299 | 7.34E-04 |
| PFC | SetShift | TNF signaling pathway | 0.529725 | 6.15E-03 |
| PFC | SetShift | Human papillomavirus infection | 0.3886186 | 2.64E-02 |
| PFC | SetShift | MicroRNAs in cancer | 0.4470705 | 2.83E-02 |
| 1. Nucleus accumbens is ACB, hippocampus is HF, and prefrontal cortex is PFC. | | | | |
| 2. Area under the curve (AUC) for percent preference for the large reward (CHL), response omissions (Omit), and response latency (Latency). For %CHL, a higher AUC reflects better delay tolerance (less impulsivity), whereas, for % omissions and response latency, a higher AUC reflects poorer neurobehavioral performance (greater omissions or slower responding). | | | | |
| 3. An enrichment score quantifies how much a set of genes (representing a pathway or function) is over-represented at the top or bottom of a ranked list of genes, indicating enrichment or depletion of that pathway. | | | | |
| 4. Cells with false discovery rate (FDR)-adjusted p value <0.05 were highlighted. | | | | |

| **Table S5.** Overlapping differentially expressed genes between PFOS exposure and neurobehavioral tests. | | |
| --- | --- | --- |
| **Tissue** | **Outcome** | **Genes** |
| ACB | CHL | ENSRNOG00000069434 |
| ACB | CHL | Nat8f2 |
| PFC | CHL | AC080157.1 |
| PFC | CHL | Clspn |
| ACB | Omit | ENSRNOG00000063145 |
| ACB | Omit | LOC120100310 |
| PFC | Omit | AC080157.1 |
| PFC | Omit | Abcg3 |
| PFC | Omit | ENSRNOG00000063145 |
| PFC | Omit | ENSRNOG00000069434 |
| PFC | Omit | Ggt6 |
| PFC | Omit | Hmgb2 |
| PFC | Omit | LOC120100310 |
| 1. Nucleus accumbens is ACB, hippocampus is HF, and prefrontal cortex is PFC. | | |
| 2. Area under the curve (AUC) for percent preference for the large reward (CHL), response omissions (Omit), and response latency (Latency). For %CHL, a higher AUC reflects better delay tolerance (less impulsivity), whereas, for % omissions and response latency, a higher AUC reflects poorer neurobehavioral performance (greater omissions or slower responding). | | |

| **Table S6.** Mediation analysis of the differentially expressed genes (DEGs) in the effect of PFOS on different neurobehavioral tests assuming independent mediated effect. | | | | | |
| --- | --- | --- | --- | --- | --- |
| **Tissue^1^** | **Outcome^2^** | **Mediator** | **Effect Type** | **beta [95% CI]** | **p value^3^** |
| ACB | CHL | ENSRNOG00000069434 | Average Mediated Effect | -4.39 [-16.29, 5.82] | 0.38 |
| ACB | CHL | ENSRNOG00000069434 | Average Direct Effect | -5.74 [-18.84, 7.9] | 0.43 |
| ACB | CHL | ENSRNOG00000069434 | Total Effect | -10.13 [-20.88, 1.22] | 0.07 |
| ACB | CHL | ENSRNOG00000069434 | Proportion Mediated | 0.39 [-1.68, 3.34] | 0.43 |
| ACB | CHL | Nat8f2 | Average Mediated Effect | -12.76 [-26, -2.51] | **0.01** |
| ACB | CHL | Nat8f2 | Average Direct Effect | 2.81 [-10.11, 16.36] | 0.68 |
| ACB | CHL | Nat8f2 | Total Effect | -9.95 [-20.61, 1.66] | 0.08 |
| ACB | CHL | Nat8f2 | Proportion Mediated | 1.19 [-3.73, 5.56] | 0.09 |
| PFC | CHL | AC080157.1 | Average Mediated Effect | -8.81 [-18.98, -0.26] | **0.04** |
| PFC | CHL | AC080157.1 | Average Direct Effect | 1.01 [-4.23, 6.22] | 0.75 |
| PFC | CHL | AC080157.1 | Total Effect | -7.8 [-17.95, 2.55] | 0.11 |
| PFC | CHL | AC080157.1 | Proportion Mediated | 1.07 [-0.62, 4.01] | 0.09 |
| PFC | CHL | Clspn | Average Mediated Effect | -7.37 [-17.54, -0.11] | 0.05 |
| PFC | CHL | Clspn | Average Direct Effect | -0.68 [-12.54, 10.45] | 0.91 |
| PFC | CHL | Clspn | Total Effect | -8.05 [-18.58, 1.78] | 0.11 |
| PFC | CHL | Clspn | Proportion Mediated | 0.84 [-3.1, 6.15] | 0.15 |
| ACB | Omit | ENSRNOG00000063145 | Average Mediated Effect | 3.69 [-2.43, 9.98] | 0.22 |
| ACB | Omit | ENSRNOG00000063145 | Average Direct Effect | -0.18 [-7.05, 6.88] | 0.96 |
| ACB | Omit | ENSRNOG00000063145 | Total Effect | 3.5 [-0.48, 7.58] | 0.09 |
| ACB | Omit | ENSRNOG00000063145 | Proportion Mediated | 0.94 [-4.84, 10.31] | 0.29 |
| ACB | Omit | LOC120100310 | Average Mediated Effect | 2.23 [-1.06, 6.35] | 0.19 |
| ACB | Omit | LOC120100310 | Average Direct Effect | 1.27 [-3.73, 6.01] | 0.6 |
| ACB | Omit | LOC120100310 | Total Effect | 3.5 [-0.37, 7.12] | 0.08 |
| ACB | Omit | LOC120100310 | Proportion Mediated | 0.59 [-2.06, 3.65] | 0.26 |
| PFC | Omit | AC080157.1 | Average Mediated Effect | 0.85 [-1.38, 3.59] | 0.46 |
| PFC | Omit | AC080157.1 | Average Direct Effect | 3.82 [-0.28, 7.78] | 0.06 |
| PFC | Omit | AC080157.1 | Total Effect | 4.67 [0.89, 8.52] | **0.02** |
| PFC | Omit | AC080157.1 | Proportion Mediated | 0.15 [-0.41, 1.06] | 0.47 |
| PFC | Omit | Abcg3 | Average Mediated Effect | 3.76 [0.59, 7.29] | **0.02** |
| PFC | Omit | Abcg3 | Average Direct Effect | 0.99 [-0.35, 2.29] | 0.14 |
| PFC | Omit | Abcg3 | Total Effect | 4.74 [1.64, 8.14] | **0.01** |
| PFC | Omit | Abcg3 | Proportion Mediated | 0.79 [0.36, 1.1] | **0.01** |
| PFC | Omit | ENSRNOG00000063145 | Average Mediated Effect | 3.13 [0.47, 6.83] | **0.02** |
| PFC | Omit | ENSRNOG00000063145 | Average Direct Effect | 1.56 [-1.33, 4.52] | 0.31 |
| PFC | Omit | ENSRNOG00000063145 | Total Effect | 4.69 [1.07, 8.31] | **0.01** |
| PFC | Omit | ENSRNOG00000063145 | Proportion Mediated | 0.65 [0.15, 1.57] | **0.02** |
| PFC | Omit | ENSRNOG00000069434 | Average Mediated Effect | 2.34 [-0.8, 6.28] | 0.16 |
| PFC | Omit | ENSRNOG00000069434 | Average Direct Effect | 2.4 [-2.36, 6.77] | 0.31 |
| PFC | Omit | ENSRNOG00000069434 | Total Effect | 4.75 [0.89, 8.32] | **0.02** |
| PFC | Omit | ENSRNOG00000069434 | Proportion Mediated | 0.48 [-0.22, 2.26] | 0.17 |
| PFC | Omit | Ggt6 | Average Mediated Effect | 0.9 [-1.95, 4.38] | 0.52 |
| PFC | Omit | Ggt6 | Average Direct Effect | 3.72 [-1.02, 7.82] | 0.12 |
| PFC | Omit | Ggt6 | Total Effect | 4.62 [0.74, 8.43] | **0.02** |
| PFC | Omit | Ggt6 | Proportion Mediated | 0.17 [-0.69, 1.42] | 0.54 |
| PFC | Omit | Hmgb2 | Average Mediated Effect | 0.56 [-3.04, 4.37] | 0.76 |
| PFC | Omit | Hmgb2 | Average Direct Effect | 4.1 [-0.8, 8.78] | 0.11 |
| PFC | Omit | Hmgb2 | Total Effect | 4.66 [0.96, 8.21] | **0.01** |
| PFC | Omit | Hmgb2 | Proportion Mediated | 0.11 [-0.95, 1.32] | 0.76 |
| PFC | Omit | LOC120100310 | Average Mediated Effect | 1.9 [-0.48, 5.35] | 0.12 |
| PFC | Omit | LOC120100310 | Average Direct Effect | 2.65 [-1.45, 6.49] | 0.18 |
| PFC | Omit | LOC120100310 | Total Effect | 4.55 [0.71, 8.39] | **0.02** |
| PFC | Omit | LOC120100310 | Proportion Mediated | 0.4 [-0.16, 1.64] | 0.13 |
| 1. Nucleus accumbens is ACB, hippocampus is HF, and prefrontal cortex is PFC. | | | | | |
| 2. Area under the curve (AUC) for percent preference for the large reward (CHL), response omissions (Omit), and response latency (Latency). For %CHL, a higher AUC reflects better delay tolerance (less impulsivity), whereas, for % omissions and response latency, a higher AUC reflects poorer neurobehavioral performance (greater omissions or slower responding). | | | | | |
| 3. p values less than 0.05 were bolded. | | | | | |

| **Table S7.** Mediation analysis of the differentially expressed genes (DEGs) in the effect of PFOS on different neurobehavioral tests assuming parallel mediated effect. | | | | |
| --- | --- | --- | --- | --- |
| **Tissue^1^** | **Outcome^2^** | **Effect** | **beta [95% CI]** | **p value^3^** |
| ACB | CHL | Total Natural Direct Effect | 25.38 [-56.86, 203.13] | 0.21 |
| ACB | CHL | Total Natural Indirect Effect | -5.56 [-100.29, 17.79] | 0.38 |
| ACB | CHL | Total Effect | -6.97 [-25.32, 3.97] | 0.14 |
| ACB | Omit | Total Natural Direct Effect | 4.08 [-19.73, 40.16] | 0.22 |
| ACB | Omit | Total Natural Indirect Effect | 5.2 [-34.72, 26.7] | 0.79 |
| ACB | Omit | Total Effect | 3.26 [-3.3, 10.27] | 0.16 |
| PFC | CHL | Total Natural Direct Effect | 3.67 [-13.92, 16.02] | 0.28 |
| PFC | CHL | Total Natural Indirect Effect | -5.4 [-33.58, 20.21] | 0.77 |
| PFC | CHL | Total Effect | -9.38 [-19.38, 4.34] | 0.16 |
| PFC | Omit | Total Natural Direct Effect | 16.73 [-93.27, 133.27] | 0.21 |
| PFC | Omit | Total Natural Indirect Effect | -17.55 [-74.54, 99.53] | 0.38 |
| PFC | Omit | Total Effect | 14.01 [-5.07, 18.62] | 0.14 |
| 1. Nucleus accumbens is ACB, hippocampus is HF, and prefrontal cortex is PFC. | | | | |
| 2. Area under the curve (AUC) for percent preference for the large reward (CHL), response omissions (Omit), and response latency (Latency). For %CHL, a higher AUC reflects better delay tolerance (less impulsivity), whereas, for % omissions and response latency, a higher AUC reflects poorer neurobehavioral performance (greater omissions or slower responding). | | | | |
| 3. p values less than 0.05 were bolded. | | | | |
